# Role of Immune Escape Mechanisms in Hodgkin's Lymphoma Development and Progression: A Whole New World with Therapeutic Implications

**DOI:** 10.1155/2012/756353

**Published:** 2012-08-15

**Authors:** Luis de la Cruz-Merino, Marylène Lejeune, Esteban Nogales Fernández, Fernando Henao Carrasco, Ana Grueso López, Ana Illescas Vacas, Mariano Provencio Pulla, Cristina Callau, Tomás Álvaro

**Affiliations:** ^1^Clinical Oncology Department, Hospital Universitario Virgen Macarena, 41009 Sevilla, Spain; ^2^Molecular Biology and Research Section, Hospital de Tortosa Verge de la Cinta and IISPV, URV, 43201 Reus, Spain; ^3^Radiotherapy Department, Hospital Universitario Virgen Macarena, 41009 Sevilla, Spain; ^4^Clinical Oncology Department, Hospital Universitario Puerta de Hierro Majadahonda, 28222 Madrid, Spain; ^5^Pathology Department, Hospital de Tortosa Verge de la Cinta and IISPV, URV, 43201 Reus, Spain

## Abstract

Hodgkin's lymphoma represents one of the most frequent lymphoproliferative syndromes, especially in young population. Although HL is considered one of the most curable tumors, a sizeable fraction of patients recur after successful upfront treatment or, less commonly, are primarily resistant. This work tries to summarize the data on clinical, histological, pathological, and biological factors in HL, with special emphasis on the improvement of prognosis and their impact on therapeutical strategies. The recent advances in our understanding of HL biology and immunology show that infiltrated immune cells and cytokines in the tumoral microenvironment may play different functions that seem tightly related with clinical outcomes. Strategies aimed at interfering with the crosstalk between tumoral Reed-Sternberg cells and their cellular partners have been taken into account in the development of new immunotherapies that target different cell components of HL microenvironment. This new knowledge will probably translate into a change in the antineoplastic treatments in HL in the next future and hopefully will increase the curability rates of this disease.

## 1. Introduction

The hallmarks of HL are mononuclear Hodgkin's cells and multinuclear Reed-Sternberg (H/RS) cells, which usually account for only 1% of cells in tumor tissue. Evidence has accumulated that H/RS cells harbor clonally rearranged and somatically mutated immunoglobulin genes, indicating their derivation, in most cases, from germinal center (GC) B cells [1–3]. Some HL cases have been identified in which the H/RS is of T-cell origin but these are rare, accounting for 1-2% of cHL. Under normal conditions, GC B cells, that lack a functional high affinity antibody, undergo apoptosis in the germinal center. H/RS cells show a characteristically defective B-cell differentiation program, lose the capacity to express immunoglobulin, and, therefore, should die. However, H/RS cells escape apoptosis and instead proliferate, giving rise to the tumor and the immune response that characterizes [1–3]. The presence of a characteristic inflammatory microenvironment is a fundamental component of the tumor mass and an essential pathogenetic factor in classical HL (cHL). It could supply the tumor cells with growth factors and could also inhibit antitumor immune responses. As the tumor cells and the reactive infiltrate grow up together, there is an extensive crosstalk between these two components mediated by cytokines and chemokines expressed by both cells. The most relevant mechanisms of immune escape are exerted by neoplastic cells but also by specific immune cells polarized towards a Th2 phenotype in order to evade antitumor immunity. The pathogenetic role of Epstein-Barr virus (EBV) potentially based on cytotoxic T cells specifically directed towards EBV antigens also appears to influence the composition of the infiltrating immune cells population, which on the other side may have an impact on clinical presentation and outcome.

The functional role of the microenvironment and the EBV in the pathophysiology and immune escape mechanisms of HL is an exciting new field of basic and translational research. Although chemotherapy and radiotherapy remain the cornerstone of HL treatment, up to 30% and 10% of patients will recur and die of HL in advanced and early disease, respectively. Therefore, current cancer research in HL aims to develop methods to increase the effectiveness of host antitumoral immune response, mainly with biologic therapies that use the body's immune system, either directly or indirectly, to fight HL.

## 2. Microenvironment Composition in HL

### 2.1. Recruitment of HL Microenvironment

In most HL cases, H/RS cells represent the minority of the tumor burden and are dispersed among reactive elements comprising mixture of inflammatory cells, stromal cells, and a predominance of Th2 cells between the various subpopulations of lymphoid cells [4, 5]. Polarized Th1 and Th2 cells represent two subgroups of helper T cells that not only exhibit different functional properties but also show the preferential expression of some activation markers and distinct transcription factors. On the contrary to Th1 cells, the Th2 cells produce IL-4, IL-5, IL-10, and IL-13, which are responsible for strong antibody production and inhibition of several macrophage functions, thus providing phagocyte-independent protective responses. In such a setting, the “pressure” of the microenvironment over the neoplastic cells may be perceived as well as a strong reciprocal influence between H/RS cells and the diverse types of reactive cells. H/RS cells have a major role in the orchestration of the microenvironment milieu associated with HL. They can directly induce the recruitment of several immune cell types from the peripheral circulation and also trigger the local expansion of diverse cellular subsets. A whole plethora of soluble mediators synthesized by H/RS cells with chemotactic activity such as the cytokines and chemokines IL-5, IL-8, IL-9, CCL-5, and CCL-28 are involved in the recruitment of granulocytes, mast cells, and macrophages, whereas IL-7, CCL-5, CCL-17, CCL-20, and CCL-22 were effectors of lymphocyte recruitment and expansion [6]. Recruitment of infiltrating immune cells is also boosted by reactive cells themselves and particularly by macrophages and mast cells synthesizing CCL-3, CCL-4, and CCL-8 chemokines [6, 7].

Chemokine receptors, CXCR3, CXCR4, and CCR7, and adhesion molecules including CD62 ligand were found to be expressed on most T cells within HL tissues, while the corresponding ligands were expressed on malignant cells and vascular endothelium. These features resemble the mechanisms of T-cell recruitment observed in normal lymph nodes, thus further highlighting the crosstalk among neoplastic and nonneoplastic cells within the HL microenvironment [8].

### 2.2. Microenvironmental Cell Types

**Figure 1:**
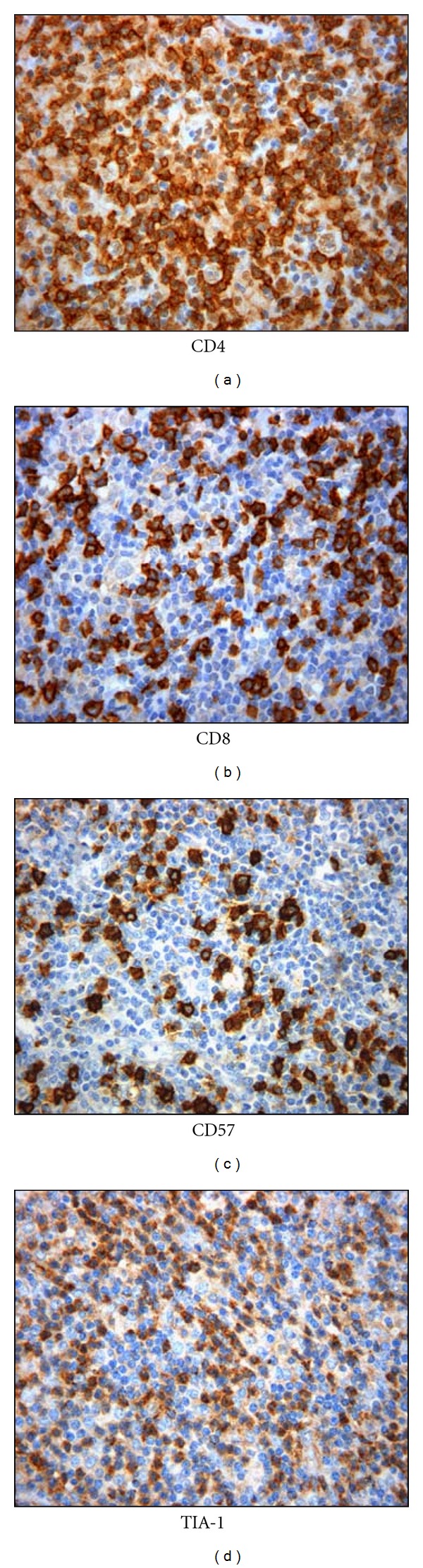
Immunohistochemical staining of inflammatory background in HL: T lymphocytes (CD4 and CD8), NK cells (CD57), and cytotoxic cells (TIA-1).

Innate immunity is represented essentially by dendritic cells (DCs), macrophages, natural killer (NK), NK/T cells, neutrophils, cytokines, and complement proteins, whereas adaptive immune cells are represented by B lymphocytes, CD4^+^ T-helper lymphocytes, and CD8^+^ cytotoxic lymphocytes (CTL). In the majority of HL tissues, different studies confirmed the predominance of CD4^+^ T lymphocytes in the background of tumoral cells in addition to a high number of cytotoxic cells (CD8, CD57, TIA-1) (Figure 1) [9–11]. The composition of the infiltrate has been seen to differ depending on the histological subtypes of cHL and the discrete stages of the disease course but also on the state of immunosuppression of HL patients. The reactive background is most pleomorphous in HL cases of the mixed cellularity histotype (MCHL), where inflammatory elements efface the lymph node architecture, while it is mainly composed of lymphocytes organized within preserved or regressed lymphoid follicles in cases of the lymphocyte-rich type (LRHL) [12–17]. In the nodular sclerosis variant (NSHL), the presence of a prominent mixed inflammatory background may be progressively reduced by the accumulation of collagen fibrosis suggesting a dynamic process of tissue remodeling [12–17].

### 2.3.  Crosstalk between Tumoral and Immune Cells

The continuous interaction pathways of H/RS cells with nonmalignant reactive and stromal cells in lymphoma tissues is now a clear evidence [6]. Several observations indicate that H/RS cells are dependent on survival signals received from immune/inflammatory cells [18]. CD4^+^ T cells, the largest population of infiltrating immune cells, are presumably particularly important [19]. Some of the survival signals that are provided by inflammatory cells to the H/RS cells are the triggering of CD40 signaling by CD40L-expressing rosetting T cells, activation of TACI and BCMA through production of their ligand APRIL by neutrophils, and perhaps activation of CD30 through CD30L-expressing mast cells and eosinophils. Moreover, H/RS cells express IL-3R, which has growth- and survival-promoting effects following activation, and there is evidence that H/RS cells can induce activated T cells to secrete IL-3 [20]. H/RS cells stimulate fibroblasts through various factors (e.g., TNF*α*, transforming growth factor-*β* (TGF-*β*), and fibroblast growth factors) [21, 22], and the activated fibroblasts in turn produce eotaxin and CCL5, thus contributing to the attraction of eosinophils and Tregs into the lymphoma [22]. H/RS cells also orchestrate their cellular microenvironment to evade an attack by cytotoxic T cells or natural killer cells. The presence of a large population of Tregs in the HL microenvironment is presumably established not only by the chemokine-mediated attraction of such cells but also by induction of differentiation of naive CD4^+^ T cells into Treg cells by H/RS cells [23]. Unexpectedly, high numbers of Treg cells in the HL microenvironment have been linked to a good prognosis, indicating that Treg cells may also have some suppressive activity on H/RS cells or on other inflammatory cells that support H/RS cell survival and/or proliferation [24, 25]. H/RS cells may further modulate their cellular microenvironment by shifting a Th1-type response to a Th2 response, which often has tumor-promoting activities [26]. H/RS cells also produce the immunosuppressive cytokines IL-10 and TGF-*β*, and galectin 1 (Gal-1) and prostaglandin E2, which inhibit T-cell effectors functions [27–31]. Moreover, T-cell effectors functions are inhibited by binding of programmed cell death protein 1 (PD-1) on T cells to the PD1 ligand that is expressed by H/RS cells [32, 33].

### 2.4. Microenvironment, Hematopoiesis, and Extracellular Matrix

The hematopoietic microenvironment is constituted by a three-dimensional complex and highly organized structure (stromal cells, extracellular matrix (ECM), and cytokines/chemokines), which serves to regulate the location, proliferation, and function of the hematopoietic cells [34]. Their alterations not only have great importance in the physiopathology of some leukemia/lymphoma but also in the formation of the intratumoral cell microenvironment. ECM represents a biophysical filter that offers protection, nutrition, and cell innervation, giving way for immune response, angiogenesis, fibrosis, and tissular regeneration [35]. Its disruption supposes a functional loss for nutrition, elimination, cell denervation, regenerative capacity, and wound healing. This also causes the loss of the immune response to pathogens, toxins, and tumoral cells. HL was the first hematopoietic tumor to be characterized as having clearly aberrant nuclear factor-*κ*B (NF-*κ*B) activity that appears closely linked to the cellular interactions within the bone marrow microenvironment: direct contact of tumoral cells with the EMC, bone marrow stromal cells (BMSCs), osteoblasts, or other cellular compartments in the BM. In HL, NF-*κ*B is constitutively activated and serves as survival factor of tumoral cells [36–38]. To date, several pathways have been suggested to induce aberrant signaling in H/RS cells, including expression of Epstein-Barr virus latent membrane protein-1 (EBV-LMP-1), increased IKK activity, functional expression of receptor activator of NF-*κ*B (RANK), or ligand-independent signaling following overexpression of CD30 [39, 40]. EBV, similarly to other viruses and certain bacteria, may induce pathological changes by epigenetic reprogramming of host cells. In HL, LMP1 can modulate cellular gene expression programs by affecting, via the NF-*κ*B pathway, levels of cellular microRNAs miR-146a and miR-155 [41]. Elucidation of the epigenetic consequences of EBV-host interactions (within the framework of the emerging new field of pathoepigenetics) may have important implications for therapy and disease prevention, because epigenetic processes are reversible, and continuous silencing of EBV genes contributing to pathoepigenetic changes may prevent disease development.

## 3. Hodgkin's Lymphoma in Immunosuppressed Patients

**Figure 2:**
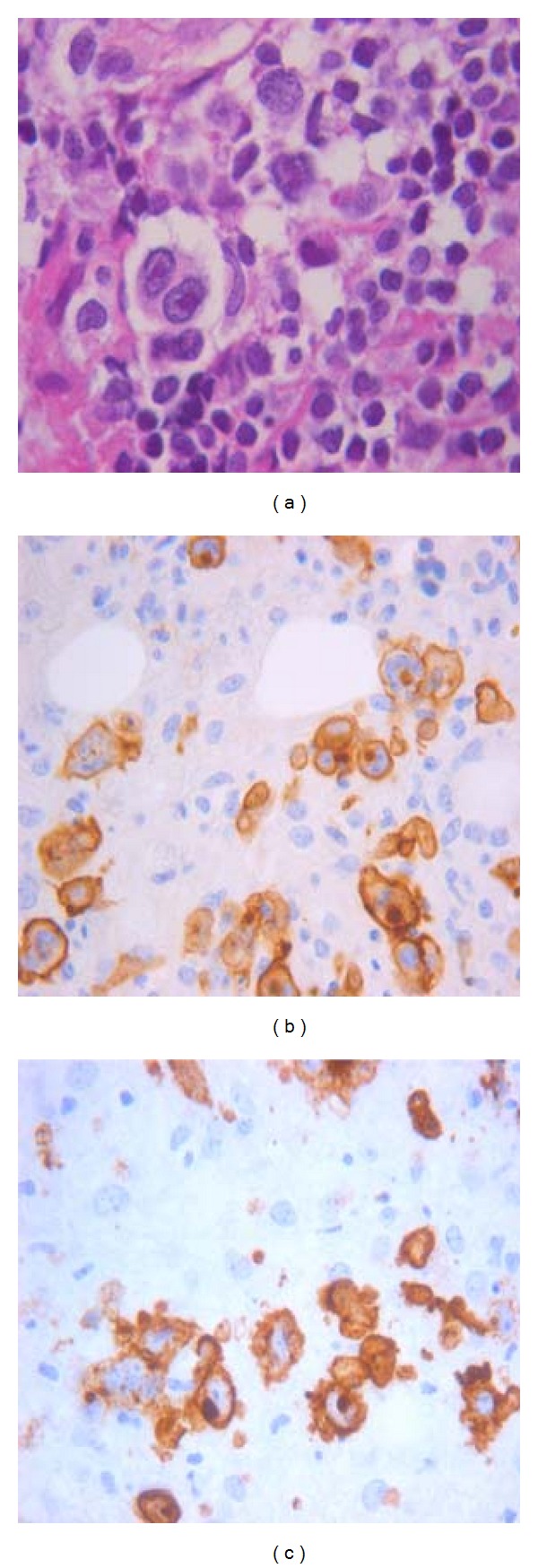
Reed-Sternberg cell (a) seen in a cellular background rich in lymphocytes of a classical Hodgkin's lymphoma. Immunohistochemical expression of the activation markers CD30 (b) and CD15 (c).

cHL in non-immunosuppressed and immunosuppressed individuals are similar in morphology of neoplastic cells, expression of activation markers such as CD30 and CD15 (Figure 2), and aberrations/activation of NF-*κ*B pathway, but they differ in the strict association with EBV infection, persistent B-cell phenotype, and CD4 cellular background composition [42]. In immunosuppressed hosts, according to the type of immunosuppression, cHL include human-immunodeficiency-virus- (HIV-) associated, iatrogenic, and posttransplant types.

### 3.1. HIV-Associated cHL

cHL represents the most common nonacquired immunodeficiency syndrome (AIDS) tumor diagnosed in patients with HIV infection. The risk is significantly increased in all ages and the risk relative to the general population ranging from 5- to 15-fold [19, 43–45]. Moreover, a significant increase in the incidence of cHL in patients treated with highly active antiretroviral therapy (HAART) has been observed. HIV-HL exhibits pathological features that are different from those of HL in ‘‘general population” with predominance of unfavorable histological subtypes (MC and LD) [46–48]. One of the peculiar clinical features of HIV-cHL is the widespread extent of the disease at presentation and the frequency of systemic B symptoms [48]. The widespread use of HAART has resulted in substantial improvement in the survival of patients with HIV infection and lymphomas because of the reduction of the incidence of opportunistic infections, the opportunity to allow more aggressive chemotherapy, and the less-aggressive presentation of lymphoma in patients in HAART compared with those lymphomas which arise in patients who never received HAART [49, 50]. Optimal therapy for HIV-cHL has not been defined yet. The widespread use of HAART allows the use of more aggressive chemotherapeutic regimens generally used in cHL in HIV negative patients. Since a large proportion of HIV-cHL progresses and relapses, the use of HDC and autologous stem cell transplantation (ASCT) has been tested in this setting [51–53].

### 3.2. Posttransplant Associated cHL

The majority of transplanted patients are initially managed by reduction and/or withdrawal of immunosuppression. Posttransplant lymphoproliferative disorders (PTLDs) are a heterogeneous group of monoclonal or polyclonal lymphoproliferative lesions that occur in immunosuppressed recipients after solid-organ or bone marrow transplantation [54, 55]. Generally, the time from transplant to the development of the disease ranges from few months (4–6 months) to several years, with a median time of 113 months, significantly longer than that of classical B-cell PTLDs. The posttransplant cHL setting, most often in renal transplant patients, is almost always EBV positive [56–59]. The distinction of Hodgkin's-like PTLD from true Hodgkin-type PTLD may be difficult [56]. Different studies have described the clinical course, generally very aggressive, and the poor outcome of patients receiving posttransplant immunosuppression. The use of chemotherapy is limited by the clinical condition of patients, and the response rate is generally lower than that observed in all other forms of cHL. Recently, rituximab has also gained favor in the treatment of PTLD because of its targeting of CD20-positive B cells, with fairly promising results [60].

### 3.3. Refractory cHL

Refractory cHL patients are defined as patients who do not respond to first-line chemotherapy or progress during treatment or relapse within 3 months after the first-line therapy. They represent 20–25% of cHL with advanced stage disease [61, 62]. Many of these patients have a poor overall survival and may die as result of their disease. To date, there is no consensus on biological markers that add value to usual parameters (which comprise the IPS) used at diagnosis to predict outcome. The prognostic significance of CD20 expression in cHL is controversial and a matter of ongoing debate [63–66]. A recent retrospective study shows that new immunohistochemical markers might predict the response to treatment of cHL based both on features of tumoral cells and on microenvironment [67]. Patients presenting either a refractory and early relapse cHL or a responding disease provided evidence that HRS cells present at diagnosis an overexpression of BCL2 marker and a frequent absence of CD20 expression and that there is an excess of cytotoxic TIA-1 and ckit-positive mast cells in the microenvironment. In patients with refractory disease, who have attained at least a partial response after salvage therapy, intensification with high-dose chemotherapy (HDC) significantly improves the outcome of patients. However, patients with primary refractory disease still showed a worse prognosis [68, 69].

## 4. Immune Escape Mechanisms in HL and Prognosis

**Figure 3:**
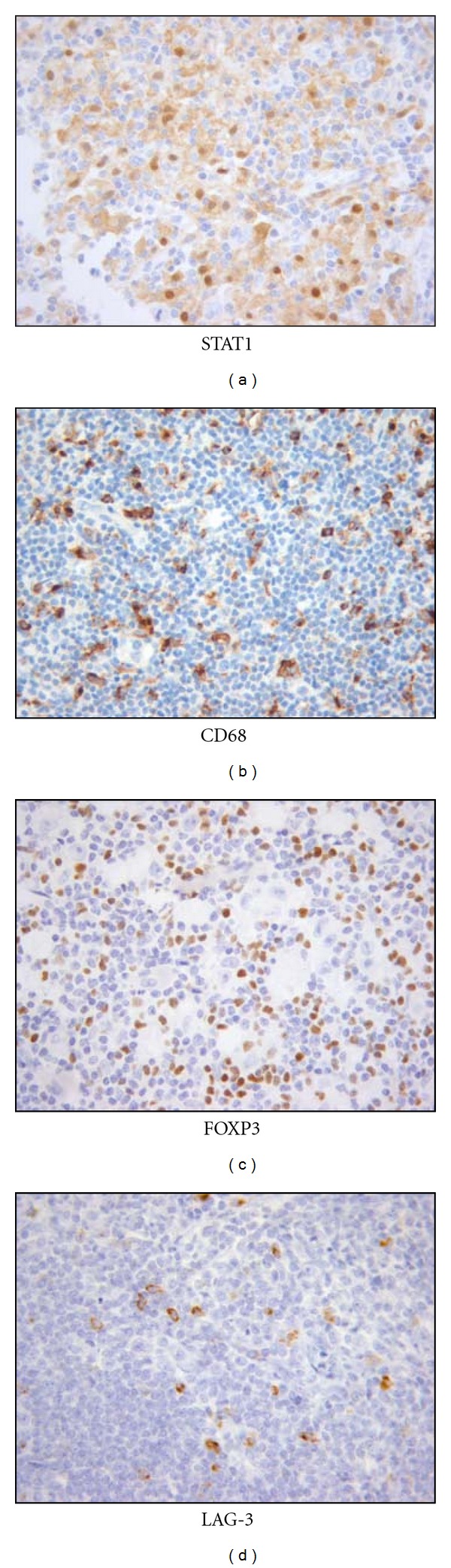
Immunohistochemical staining of immunosuppressive cells in HL: tumor-associated macrophages TAM (STAT-1 and CD68) and regulatory T cells (FOXP3 and LAG-3).

The immune system has the ability to act as a double-edged sword, indicating that tumor elimination requires a good coordination of the various elements of the immune system (Table 1). If tumoral cells employ a plethora of immunosuppressive mechanisms, which may act in concert to counteract effective immune responses, different subsets of immune cells contribute also to this immunosuppressive network (Figure 3) [70].

### 4.1. Tumoral Protective Action

The microenvironment of HL is sustained by an autocrine and/or paracrine production of several cytokines including, among others, IL-5, IL-8, IL-9, CCL-5, and CCL-28. The release of these molecules is also responsible for most of the symptoms recorded in patients with HL, in addition to the ability of the neoplastic cells to escape from growth controls and immunosurveillance. Effectively, H/RS cells are able to sense growth and survival signals coming from the growth factor milieu, owing to the expression of a broad range of receptors including IL-7R, IL-9R, IL-13R, TACI, and CCR5 [6]. Along with growth factors, also proinflammatory cytokines and mediators can sustain H/RS cell expansion through the activation of pathways converging into the NF-*κ*B focal point, such as those triggered by IL-6R, TACI, RANK, TNFR-1, Cys-LT receptors, and NOTCH-1 engagement [71]. These proinflammatory spurs may be either derived from the microenvironment (e.g., leukotriene production by mast cells and NOTCH-1 ligand expression by stromal cells) or originate from both H/RS cells and reactive elements (e.g., IL-6, TNF).

The H/RS cells secrete high amounts of chemokine, thymus and activation-regulated chemokine (TARC) and macrophages-derived chemokine (MDC) in particular, which attract lymphocytes expressing CCR4 receptor, such as Th2 [28]. These cytokines may contribute to the pathogenesis of the disease initiated and sustained the presence of the reactive infiltrate. Immune cells present in the local infiltrate have proved to be capable of modulating apoptosis and of inducing proliferation of tumoral cells via death receptors, cytotoxic granule liberation, and withdrawal of growth factors or production of immunosuppressive cytokines [28, 72–74]. In HL, it has been initially proposed that CD4^+^ T cells produce cytokines of Th2 type that could contribute to local suppression of the cellular immune response mediated by Th1 cells [75, 76]. Immunoregulatory cytokines such as IL-10 and TGF-*β* play an important role in immune tolerance, and it seems that suppressor effect of regulatory T cells (CD4^+^CD25^+^) on the development of tumor associated antigen-reactive lymphocytes is independent of cytokines [20].

Several other molecules have been tested for their possible involvement in such a context. For instance, Prostaglandin E2 has been shown to impair CD4^+^ T-cell activation [31]. Tissue inhibitor of metalloproteinases 1 (TIMP-1) is a protein with proteinase-inhibiting and cytokine properties which has been advocated not only as a survival factor for H/RS cells but as potential immunosuppressive agent. Also, the downregulatory molecule cytotoxic T lymphocyte-associated antigen 4 (CTLA-4) was shown to play a possible role, as the proportion of CTLA-4^+^/CD3^+^ cells negatively correlated with proliferative activity, IL-2 and IFN-*γ* production by T lymphocytes in HL patients [77]. Even CD30, which is typically expressed on HRSC, was shown to inhibit T cell proliferation [78]. Other potentially involved molecule is the Gal-1, produced by H/RS cells. In fact, blockade of Gal-1 was able to restore the Th1/Th2 balance [27]. It has been also proposed that hepatocyte growth factor and c-MET might constitute an additional signaling pathway between H/RS cells and the reactive cellular background, affecting adhesion, proliferation, and the survival of H/RS cells [79].

The 15–25% of HL patients who did no respond to standard chemotherapy regimens will die of relapse [80], probably due to the presence of a small number of cells resistant to chemotherapy or radiation treatment that are not eliminated by the endogenous immune system. This minor side population (SP) of tumor cells has been previously identified as cells with stem/progenitor cell-like characteristics from normal [81, 82] and malignant tissues [83–86]. These SP cells also express multidrug transporter proteins, including MDR1 and ABCG2 [87, 88], which not only efflux Hoechst dye but also rapidly reduce the intracellular concentrations and thus the cytotoxicity of many commonly used therapeutic drugs [89, 90]. In a more recent study, Schafer and collaborators identified a distinct SP subset in HL cell lines and primary tumor biopsies that are resistant to gemcitabine [91]. This SP subset also expresses tumor-associated antigens, which render them susceptible to killing by tumor-specific CTLs following demethylation with decitabine. This study suggests that combination therapeutic strategies that use conventional chemotherapy to debulk tumor burden, followed by novel drugs such as histone deacetylation (HDAC) inhibitors and T cell immunotherapy, may eliminate residual chemoresistant tumor cells and help prevent disease relapse.

### 4.2. Reprogramming of Tumoral Cells

The escape from apoptosis and transcriptional reprogramming of H/RS cells are interlinked and seem important to disease pathogenesis. In cHL and primary mediastinal B-cell lymphoma, genomic breaks of the major histocompatibility complex (MHC) class II transactivator CIITA have been demonstrated to be highly recurrent (15% and 38%, resp.) [92]. The functional consequences of CIITA gen fusions is the downregulation of surface HLA class II expression and overexpression of ligands of the receptor molecule PD-1 (CD274/PDL1 and CD273/PDL2). These receptor-ligand interactions have been shown to impact antitumor immune responses in several cancers, whereas decreased MHC class II expression has been linked to reduced tumour cell immunogenicity. The exploration of the possible role played by the PD-1 protein shows that this molecule (expressed on the surface of activated T cells, B cells, and macrophages) and its ligands are involved in the functional impairment of T cells in chronic viral infections or HL tumor immune evasion. HL was shown to overexpress PD-1 ligand, while PD-1 was markedly elevated in tumor-infiltrating and peripheral T cells of these patients. Moreover, blockade of the PD-1 system was able to restore the IFN-*γ* production by HL-infiltrating T cells [33]. Using a genome-wide transcriptional approach, CD4^+^ T cells in HL were demonstrated to be under the inhibitory influence of both TGF-*β* and PD-1 in vivo [32]. An increase in the number of PD-1^+^ lymphocytes, measured within a tissue microarray platform, was also shown to be a stage-independent negative prognostic factor of overall survival as opposed to the number of FOXP3^+^ Tregs [93]. All these findings seem to suggest that the impairment of the typical immune response in HL, is, at least partially, mediated by the PD-1 signaling pathway.

### 4.3. Regulatory T Cells (Tregs)

The categorization of CD4^+^ T cells in Th1 and/or Th2 constitutes an oversimplification and it has been shown that regulatory T cells with CD4^+^CD25^+^ phenotype not only play a role in controlling autoimmunity but also have suppressive effects on immune responses [94–96]. In cancer-bearing animals or patients, Tregs expand, migrate to tumor sites, and suppress antitumor immune response mediated by NK cells, CD4^+^ and CD8^+^ T cells, and myeloid cells, through different molecular mechanisms [97]. Functional and molecular characterization of these cells has been facilitated by the identification of markers such as FOXP3 and others [98–100]. FOXP3 encodes a transcription factor known as Scurfina, specifically expressed by T cells CD4^+^CD25^+^ [101], that acts on converting naïve regulatory T cells CD4^+^CD25^−^ phenotype to CD25^+^ [102]. More recently, it was suggested that regulatory T cells and PD1^+^ T cells interact with H/RS cells [24, 33, 103], which produce the T regulatory attractant Gal-1 and the PD-1 ligand, PDL-1 [33]. On the other hand, the observation of numerous CXCR3^+^ lymphocytes in some HL tumors has raised the possibility of an occasional Th1-predominant immune response [10].

The regulatory T cells can inhibit the production of IL-2 to regulate the high expression of IL-2R*α* (CD25), that is, delay or block the activation of CD8^+^ cells and NK cells against tumor antigens [104, 105]. The immunosuppressive properties of regulatory T cells appear to be particularly important because of its large effect on cellular cytotoxicity represented by CTLs and NK cells. The presence of low numbers of FOXP3^+^ cells and a consequent high rate of TIA-1^+^ cells in the infiltrate represents an independent prognostic factor negatively affecting the survival of the disease. Furthermore, when the disease relapses and progresses, larger number of TIA-1^+^ cells and lower proportion of FOXP3^+^ on the reactive background of the tumor are also prone to be seen [24].

It has been also hypothesized that the contribution of Tregs to HL might be function of the microenvironment polarization. Indeed, Tregs may limit the inflammatory spur of other cells of the immune system (including T effectors) by releasing IL-10 and TGF-*β*, and this beneficial effect may prevail over the impairment of an effective T-cell-mediated response, as far as the outcome of HL is concerned. Nevertheless, when the HL-associated environment is diverted towards marked inflammation owing to the abundant presence of mast cells and macrophages, the regulatory function of Tregs may prove inadequate to restore the balance between pro- and antiinflammatory stimuli, and Tregs can even boost inflammation through TGF-*β* release and Th17 generation. Under these circumstances, a direct role for mast cells in the Tregs contrasuppression and Th17 deflection can be envisaged as both mast cells and Tregs populate HL infiltrated areas, and their interaction is therefore possible. In an effort to inhibit suppressive signals counteracting activation, removal of Tregs leads to effective antitumor immunity [106]. In certain solid tumor models, depletion of Tregs in combination with immunostimulatory treatments even causes rejection of already established tumors [99, 107].

### 4.4. Tumor-Associated Macrophages (TAM) and Myeloid-Derived Suppressor Cells (MDSC)

Chronic inflammation in some tissues correlates with higher risk of developing tumors [108]. Within the tumor microenvironment, tumor-associated macrophages (TAM) and myeloid-derived suppressive cells (MDSC) seem to play a critical role in the progression of tumor development through nonimmune (mostly proangiogenic) and immune mechanisms [109]. TAMs are a heterogeneous population of cells depending on oxygen availability and phases of tumor development [110]. In early stages, tumors are generally infiltrated by type 1 macrophages (M1) that release proinflammatory cytokines and chemokines promoting Th17 cell differentiation from naïve CD4^+^ T cells [111]. On the other hand, in advanced stages, TAM polarize to a type-2-macrophage- (M2-) related cell that releases cytokines such as TGF-*β*1 and IL-10, which induce Th2 differentiation and recruitment, favoring Tregs development and thus promoting tumor development through inhibition of anticancer immune responses [112]. It is now accepted that TAM, major players in the connection between inflammation and cancer, summarize a number of functions (e.g., promotion of tumor cell proliferation and angiogenesis, incessant matrix turnover, repression of adaptive immunity) which ultimately have an important impact on disease progression [113–115]. High levels of TAM are often, although not always, correlated with a bad prognosis, and recent studies have also highlighted a link between their abundance and the process of metastasis [116–119]. Macrophage infiltration began very early during the preinvasive stage of disease and increased progressively [120]. This pathological evidence has been confirmed also at gene level, where molecular signatures associated with poor prognosis in lymphomas and breast carcinomas include genes characteristic of macrophages (e.g., CD68) [121–123]. In human HL progression, macrophages are anything but innocent bystanders since the expression of CD68 showed to be the best predictive biomarker for risk stratification and survival for this type of cancer. A high number of CD68^+^ cells correlates with primary and secondary treatment failure [124]. Another report suggested that TAMs in HL subtypes might differ in their expression of inflammatory and matrix-remodeling genes [125].

Recent studies in model animal suggest that macrophages were responsible for restoring tumor vascularisation and repair after irradiation [136]. In effect, after irradiation, the remaining tumor mass sends tissue damage signals to initiate repair, which includes the recruitment of macrophages to the tumor, aiding in the recovery of tumor growth by enhancing angiogenesis, supplying growth factors, and creating a local immunosuppressive environment. Treatment of tumors with antibodies specific for CD11b can block macrophages recruitment and inhibit tumor regrowth and survival [137]. In the same way, the use of clodronate, a liposome toxic to phagocytes, or the use of Enbrel, a blocking against TNF-*α*, inhibits tumor recovery after irradiation [136]. The detrimental contribution of mast cells and CD68^+^ macrophages to HL patients' survival has been clearly established and has been linked to the ability of both types of cells to induce and maintain the aforementioned proinflammatory microenvironment [124, 138, 139].

### 4.5. Cytotoxic T-Cells Inhibition

Different mechanisms have been suggested to account for the CTL-mediated apoptosis resistance of H/RS cells, such as the downregulation of MHC class I molecules of the H/RS cells, prevention of recognition of tumor-associated antigens by CTLs [126], or the local secretion of both IL-10 and TGF-*β* by H/RS cells [127, 128], which are able to inhibit CTL function. In this respect, it appears that the blockage of the Granzyme B pathway of apoptosis through the overexpression of serine protease inhibitor PI-9/SPI-6 is an important additional mechanism for immune escape by tumors [129]. The expression of PI9 tends to be associated with a high percentage of activated CTLs, especially in HL [75], explaining why tumors expressing high levels of PI9 have a particularly poor clinical outcome.

LAG-3 was found strongly expressed on Tregs present in the proximity of H/RS cells and the proportion of LAG-3-expressing lymphocytes correlated with the EBV status of the tumor [130]. The level of LAG-3 expression on the Tregs was coincident with impairment of LMP1/2-specific T-cell function [130] suggesting a pivotal role for LAG-3^+^ regulatory T cells in the suppression of EBV specific cytotoxic CD8^+^ cell-mediated immunity in HL [130]. It has been suggested that LMP1- and EBNA1-specific HLA class II-restricted peptide epitopes can selectively recruit regulatory T cells and impair antigen-induced IFN-*γ* production [131, 132]. LAG-3 has high affinity for MHC class II molecules and downregulates CD3 T-cell receptor mediated signaling and blockade of LAG-3 mediated signaling induces enhanced activation of human CD8 T cells [133–135]. Preliminary results of EBV specific CTL therapy in relapsed/refractory EBV-positive HL patients are encouraging [140] and, taken together, these findings have important implications in the improved design of immunotherapeutic strategies to boost LMP1/2-specific CTL activity.

## 5. New Molecular Prognostic Parameters versus Traditional Clinicobiological Prognostic Parameters

A huge amount of clinical and biological factors have been related with the risk of relapse and progression in HL, and consequently with the therapeutical strategy planned in every single patient. Recent contributions determine that HL represents the prototypical tumor in which the interplay between H/RS and the reactive microenvironment determines not only the histological morphology and classification but also the clinicopathological features and prognosis of these patients [141].

### 5.1. Traditional Clinicobiological Parameters

Similar to other lymphomas, nowadays selection of treatments in HL continues to depend on initial risk stratification. In this sense, stage remains the single most important factor in the initial approach for treatment of HL, being the Ann Arbor's system with Cotswolds modifications the current staging system used for patients with HL [142].

In clinical practice, HL is classified in early and advanced disease [143]. Early disease includes stages I-II and it is generally divided into favorable and unfavorable categories based upon the presence or absence of certain clinical features, such as age, erythrocyte sedimentation rate (ESR), B symptoms, and large mediastinal adenopathy. Cooperative research groups have used diverse definitions of favorable and unfavorable prognosis disease [144]. This stratification is highly pertinent and useful since patients with favorable prognosis disease may have acceptable outcomes with less intensive therapy than that required for those with unfavorable prognosis early stage or advanced stage disease [145].

Among patients with advanced stage HL (stage III/IV, and for some groups stage II plus bulky nodal disease), prognosis is largely determined by the International Prognostic Score (IPS) [146]. The IPS was created by the IPS Project on Advanced Hodgkin's Disease after analyzing several possible prognostic factors in 1,618 patients that were treated mainly with ABVD-like regimens. Finally, the IPS is based upon the total number of seven potential unfavorable features at diagnosis: serum albumin less than 4 g/dL, hemoglobin less than 10.5 g/dL, male gender, age over 45 years, stage IV disease, white blood cell count ≥15,000/microL, and lymphocyte count less than 600/microL and/or less than 8 percent of the white blood cell count. All of these adverse prognostic factors were statistically significant at the multivariate analysis. Patients with four or more adverse features had a significantly inferior freedom from progression (47% versus 70%) and overall survival (59% versus 83%). Coupled with stage, the IPS allows identification of a poor-risk group of patients requiring more intensive therapy [146]. Consequently, different treatment policies are indicated upon the presence of these clinicobiological parameters, with application of more aggressive approaches when more risk factors are present.

### 5.2. Innovative Biologic Prognostic Parameters

**Figure 4:**
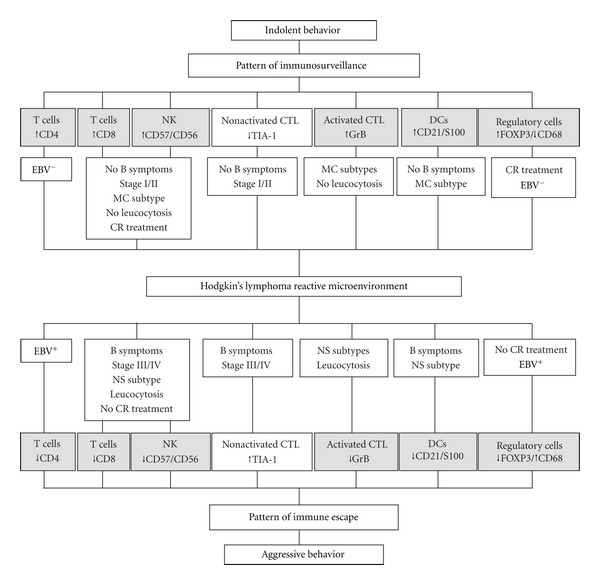
Representation of the two immune patterns observed in HL significantly associated with their clinicopathological features. The immunosurveillance pattern with a high proportion of infiltrating T lymphocytes, NK cells, DCs, activated CTL, but low proportion of resting CTL and TAM is associated with a favorable outcome. The immune escape pattern with a high proportion of infiltrating resting CTL and TAM, but low proportion of T lymphocytes, NK cells, DCs, and activated CTL is associated with an unfavorable outcome. MC, mixed cellularity; NS, nodular sclerosis; CR, complete response.

Current predictive systems, determined by clinical and analytical parameters, fail to identify high-risk patients accurately (patients who relapse or die). Quantitative analysis of infiltrating immune cells reveals undisclosed relationships between the relative proportion of these cells and HL clinical outcome, illustrating how factors other than tumoral cellularity, or the immunophenotype and molecular anomalies present in the H/RS cells, can play a role in tumoral behavior. Regardless of the classic clinical and pathological features, a high proportion of infiltrating CD8^+^ and CD57^+^ cells as well as a low number of infiltrating CTL (evaluated by the presence of Granzyme B and TIA-1) appear to be associated with a favorable outcome for HL patients (without B symptoms and lower clinical stages) and better response to treatment [10, 24, 147]. It is unclear to date whether the presence of CD8^+^ T cells correlates with the antitumor cytotoxic response. Nevertheless, it has been suspected that CD8^+^ T cells may be recruited in an antigen-nonspecific mode in HL (Figure 4) [148].

A multistep approach to design a quantitative PCR assay has been applied to routine formalin-fixed paraffin-embedded sample integrated genes expressed by the tumor and their microenvironment [149]. In cHL with advanced stage, specific gene signatures associated with favorable or unfavorable clinical outcome have been identified. The best predictor genes were integrated into an 11-gene model, including 4 functional pathways: cell cycle (CCNA2, CDC2, HMMR, CCNE2, CENPF), apoptosis (BCL2, BCL2L1, CASP3), macrophage activation (LYZ, STAT1), and interferon regulatory factor 4. These genes are able to identify low- and high-risk patients with different rates of 5-year failure-free survival: 74% versus 44.1% in the estimation set and 67.5% versus 45.0% in the validation set.

Although the activation status of infiltrating cells have been demonstrated to be independent of the degree of malignancy in HL [150], others studies have shown that the presence of activated cytotoxic T cells (granzyme B^+^) is associated with unfavorable followup in these patients [11, 151, 152]. A higher level of not activated cytotoxic cells (TIA-1^+^) has been observed in advanced-stage cHL without prognostic value [153]. However, TIA-1^+^ CTL associated with the presence of regulatory T cells FOXP3^+^ appears to play an important role in monitoring HL patients [24]. Variations in the level of killer cells and TIA-1^+^ regulatory T cells observed during the course of the disease could be implicated in the progression of HL [24].

Association of tumor-associated macrophages (TAM) CD68^+^ with adverse clinical outcomes has been confirmed in several studies in hematologic and solid tumors [139]. Recently, a gene expression profile analysis performed on 130 biopsy samples from HL patients identified a signature of TAM and monocytes that was predictive of treatment failure [124]. When compared with those with low CD68 expression, patients with tumors that demonstrated an increased number of CD68 expressing macrophages had shorter median progression-free survival (PFS), lower rate of 10-year disease-specific survival (60 versus 89%), and higher failure rate of secondary treatment with curative intent (63 versus 13%). It has been also recently demonstrated that high level of CD68 correlated with poorer survival, event-free survival, and with the presence of EBV in the tumor cell population [154].

Biologic markers associated with apoptosis/proliferation have been also studied in HL. Different studies have described alterations in genes controlling apoptosis and proliferation of H/RS cells and biological factors such as EBV detection, which influence the clinical aggressiveness of the disease [155–165]. Shorter survival was significantly associated with high proliferation index (Ki67), high expression of bcl2, bcl-xl, bax and p53, low expression of Rb and caspase 3, and high apoptotic index [163, 166–173]. Evidence has accumulated that the constitutive activation of the NF-*κ*B pathway in H/RS cells is of particular importance for explaining the apoptosis deregulation in cHL [157, 158, 160, 174]. By gene expression profiling, the good outcome cHL was characterized by upregulation of genes involved in apoptosis induction and cell signaling, including cytokines and transduction molecules, while the bad outcome cHL were characterized by upregulation of genes involved in cell proliferation (Ki67) and by downregulation of tumor suppressor genes PTEN (phosphatase and tensin homolog deleted on chromosome 10) and DCC (deleted in colorectal cancer) [175].

Immune cells present in the infiltrate have been shown to modulate the apoptosis and proliferation of tumor cells via apoptotic receptors, cytotoxic granule release, growth factors, or immunosuppressive cytokines [72–74, 176, 177]. IHC study has demonstrated that the antiapoptotic profile observed in H/RS cells is associated with a general increase in CD4^+^ T cells infiltrating (related to Bcl-XL and Mcl-1) and an overall decline CD8^+^ T lymphocytes infiltrating, NK cells, and dendritic cells (related to Bcl-XL and Bax) [178]. The infiltrated immune cells are able to activate apoptotic caspase proteolytic cascade through TNF receptor superfamily interactions (FasL/Fas and CD40/CD40L) [158, 179–183]. CTLs are also able to trigger a second proapoptotic pathway through the protease granzyme B, which, once released from CTLs, is translocated into the target cell by perforin, where it activates the effector caspase cascade [184].

Alterations observed in the G1-S checkpoint of H/RS cell cycle, in the principal tumor suppressor pathways Rb-p16INK4a and p27KIP1, and the high rate of proliferation (MIB1, BCL6) are also strongly associated with higher infiltration of the overall immune response against the tumor [178, 185, 186]. Cytotoxic cells are able to induce directly the permanent downregulation of p27KIP1, probably as a consequence of increased degradation mediated by SKP2, an ubiquitin ligase for p27KIP1 [186–188]. Related with the heightened proliferative state in these tumors is the high level of expression of Bcl6, a multifunctional regulator that is able not only to downregulate cyclin D2 and p27KIP1 expression [189] but also to repress Bcl-XL [190].

The presence of EBV was significantly associated with the overexpression of STAT1 and STAT3. STAT3 was found to be associated with a low infiltration of CD4 T lymphocytes and a high infiltration of activated cytotoxic cells. Although STAT1 is considered to be a potential tumor suppressor (promoting apoptosis), STAT3 is thought to be an oncogene because it leads to the activation of cyclin D1 and Bcl-XL expression and is involved in promoting cell cycle progression and cellular transformation and in preventing apoptosis [191].

## 6. Impact of Viruses Infection in HL Microenvironment

Early epidemiologic data suggested that HL develops among persons with a delayed exposure to a ubiquitous infectious agent such as EBV [192]. EBV, a *γ* herpesvirus with a worldwide distribution, is present in H/RS cells of 40%–60% of cHL lesions and contributes to their pathogenesis [193, 194]. EBV^+^ H/RS cells express LMP1, LMP2A, LMP2B, the EBV nuclear antigens 1 (EBNA1), and the EBER RNAs, but consistently lack EBNA2 (latency II) [195, 196]. LMP1 is likely to contribute to survival and proliferation of H/RS cells through activation of NF-*κ*B and AP-1 [197, 198]. It is also possible that EBNA1 and the EBERs contribute to the rescue of H/RS cells from apoptosis [199, 200].

The intratumoral immunological alterations induced by EBV^+^ H/RS cells remain unclear. The abnormal network of cytokines/chemokines and/or their receptors in H/RS cells is involved in the attraction of many of the microenvironmental cells into the lymphoma background. There is increasing evidence suggesting a change in the balance between Th1 and Th2 cells in the pathogenesis of HL and that this change induces reactivation of latent viral infections, including EBV. EBV-infected H/RS cells were shown to stimulate also the stromal production of particular chemokines such as the interferon-inducible chemokine IP-10 (CXCL10) [103] Rantes/CCL5 [201, 202], the ligand CCL28 [203], CCL20 that is capable of attracting regulatory T cells [204], and the macrophage-derived chemoattractant (MDC)/CCL22 [205]. The observation of Th1/antiviral response in EBV^+^ cHL tissues provides a basis for novel treatment strategies [28, 206]. The role of Gal-1, which has been shown to be selectively overexpressed on H/RS cells, was also examined in the context of EBV-specific CD8^+^ T-cell responses in HL. Its expression was associated with a reduced CD8^+^ T-cell infiltration and more specifically with an impaired response towards LMP 1 and 2. Moreover, the in vitro exposure to recombinant Gal-1 inhibited proliferation and interferon-gamma expression by EBV-specific T cells [29].

A low proportion of CD4^+^ cells appears also to be significantly related to EBV status, probably due to the relation with the local tumor-associated suppression of EBV-specific T-cell responses observed in EBV^+^ HL cases [207]. In the case of immunosuppressed patients, HIV infection affects, for direct or indirect mechanisms, both reactive changes as neoplastic lymphoid tissue. Recently we have seen a significant loss of intratumoral T cells CD4^+^ (CD4/CD8 ratio reversal) and a decrease in intratumoral activated CTL in patients with HIV-infected HL [208].

A link between septic environment, the high prevalence of Th17 cells, and the favorable outcome impact of intratumoral regulatory T cells (Tregs) has been postulated. The putative role of the dense microbiological flora present in the large intestine with a trend toward translocation through the tumor has been emphasized to explain the favorable outcome of patients bearing colorectal carcinoma (CRC) with a high Tregs infiltration [209]. This microbiological hazard requires a T-cell-mediated inflammatory antimicrobial response that involves Th17 cells. This Th17-cell-dependent proinflammatory and tumor-enhancing response can be attenuated by Tregs, thus constituting a possible explanation for their favorable role in CRC prognosis. In HL, characterization of the inflammatory cytokine profiles in EBV-HL patients revealed elevated Th2 and Th17 responses [210].

## 7. Therapeutic Strategies to Overcome Immune Escape in HL

**Figure 5:**
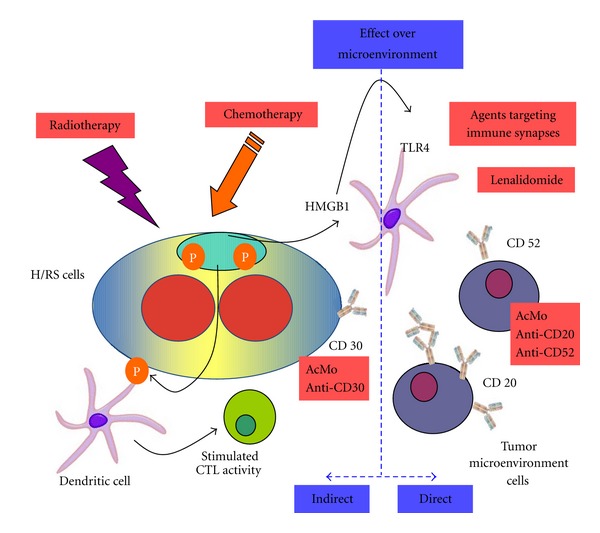
Therapeutic strategies to overcome immune escape in HL. AcMo: monoclonal antibodies. H/RS cells: Hodgkin's/Reed-Sternberg cells. CTL: cytotoxic T lymphocytes. HMGB1: high-mobility group protein B1. TLR4: Toll-like receptor 4.

In the last years, numerous studies have revealed the critical importance of the microenvironment in the evolution and progression of HL after antineoplastic treatments. This fact has opened new ways for clinical research taking into account the impact of the classical and new immunogenic agents over the characteristic Hodgkin's microenvironment, envisioning alternative treatment strategies (Figure 5).

### 7.1. Chemotherapy

Chemotherapy remains the therapeutical modality of choice for the systemic treatment of HL for curative purposes. Impact of conventional chemotherapy on the relationship between the tumor and the immune system seems to be crucial. Some groups have confirmed that cell death induced by chemotherapy imply a variety of immune reactions that mediate a sort of vaccination effect via release of an “antigenic milieu” that, in turn, may represent the major determinants of the therapeutical success of this treatment in lymphoproliferative syndromes [211]. Preclinical studies have demonstrated that immune stimulation might be mediated by chemotherapy in murine cancer models treated with gemcitabine and doxorubicin [212, 213]. The explanation to this selective immune activation is an increased CD8 T lymphocyte expansion and an increased density of TIL mediated by an effective MHC class I cross-presentation of tumor antigens released and phagocytosed [214].

Thus, there are now clear evidences supporting the fact that drugs like anthracyclines, cyclophosphamide, or gemcitabine may promote apoptosis in tumor cells with immunogenic effects through several mechanisms [215]. This sort of immunogenic tumor cell death is characterized by a temporal sequence of events including early translocation of calreticulin (CRT) to the cell surface and thereafter interaction of CRT with multiple receptors on DC with apoptotic bodies phagocytosis, release and exposure of heat shock proteins, and late release of high-mobility group protein B1 (HMGB1). HMGB1 is able to bind to the toll-like receptor 4 (TLR4) on DC, which allows tumor-derived antigens to be processed and presented along with MHC and costimulatory molecules on the surface of DC [216]. These mechanisms altogether serve to trigger DC-mediated specific antitumor response, which may be enhanced by the use of costimulatory molecules [217]. In addition, other more general effects of chemotherapy on the surrounding stroma are postulated like secondary necrosis or eradication of tumor cells [218]. Gemcitabine has demonstrated the ability to restore immune surveillance by reducing MDSC levels in murine models [219].

In conclusion, emerging evidence led to postulate a paradigm shift in the way of understanding the effects of CT on the surrounding stroma [220, 221]. These new findings may serve to consider chemotherapeutics like anthracyclines and gemcitabine as less empirical and more specific drugs, and thus may help to customize treatments in HL taking into account their potential effects on the microenvironment.

### 7.2. Radiotherapy

Radiation therapy still plays a major role in the management of HL. Ionizing radiation can induce a cascade of pro-immunogenic and proinflammatory effects. Different responses have been described: major histocompatibility complex induction, release of specific antigens, and chemokines production. All these mechanisms are triggered from the radiotherapy target area, neighborhood tissues, and also from the systemic immunological response. These inflammatory effects can convert the irradiated site into an immunogenic hub, by engaging both the innate and adaptive immune response [222, 223]. Locally ionizing radiation promotes the differentiation of monocytes into macrophages with an M1 phenotype (tumor inhibiting) and also the release of radiation-specific antigens that promotes the development of a sustained and effective adaptive immune response to the tumor [223, 224].

The immune-modulating effects of radiation are influenced by several factors. Low radiation dose activates innate immune cells and fails to induce cell death, and thus induces a protumorigenic effect over the immune system [225, 226]. Otherwise, significant radiation dose leads to cell death and induces specific signals that are sensed by innate immune cells, which generate an antitumor immunity.

Classically, apoptosis has been considered as a nonimmunogenic event. Nevertheless, as happens with anthracyclines, radiotherapy might induce apoptosis due to an immunogenic mechanism. Both therapies translocate CRL from the endoplastic reticulum to the cell surface. The superficial expression of CRL in tumor cells causes an important tumor immune response with effective recognition and phagocytosis by DC, leading to cytotoxic T-cell response. In the same manner, a cytotoxic response mediated by CD8^+^ T cells occurs when heat shock proteins (HSP) such as HSP70 and HSP90 are transferred to the plasma membrane. HSP also stimulate natural killer-mediated cell lysis, through NKG2A ligands. Secondary, DCs mature and release proinflammatory cytokines, such as HMGB1, which binds to toll-like receptor 4 (TLR4). Those mechanisms enable antigen processing and presentation [215, 227].

An accurate radiotherapy treatment has increased its interest under an immune point of view. Effectively, the reduction of naïve T-cells account after an irradiation of drainage lymphs without disease can lead to a distal reactivation of malignancies T cells [222].

The radiation abscopal effect is described as the reduction of the tumor growth outside the treatment field, and some clinical cases of this effect has been reported in different tumor types, including lymphoma [228–230]. Their mechanisms and therapeutic approach are not well elucidated [231]. However, main hypotheses imply that local irradiation induced a release of systemic cytokines that mediate an immune antitumor effect and/or the fact that local irradiation might induce systemic tumor specific T-cell responses.

Preliminary results promise that immunotherapy may serve as booster, amplifying immune effectors triggered by radiotherapy as exemplified in experiments that combine it with anti-CTLA-4 monoclonal antibodies or costimulators such as GM-CSF, interferons, or IL-2. Radiotherapy can induce cancer cell death that is mediated by the host's immune system. Elucidation of these mechanisms might offer advantages in the cytotoxic therapy.

### 7.3. Immune Synapses as Therapeutic Target in HL

The immune synapse is a region of physical contact between the T cell and the antigen presenting cell (APC) and it represents one of the major determinants of the immune response against tumoral antigens [232]. Two main signals are required for an effective T-cell activation. The first signal is provided by the recognition of cognate antigen bound major histocompatibility complex (MHC) by the T-cell receptor (TCR) [233]. Additional costimulatory signals are provided by engagement of coreceptors. The canonical coreceptor CD28 binds to members of the B7 family present on APC. However, soon after T-cell priming, other negative regulatory molecules are induced on T-cells leading to downregulation of the T-cell response [234]. Some of the main molecules that act as immune checkpoints on the immune synapse are CD40 and OX40 with costimulatory properties and CTLA-4 and PD1 that induce coinhibitory effects. Preclinical data support an eventual role of the drugs targeting these molecules in HL.

CTLA-4 acts as a key negative regulator of CD28 dependent T-cell activation [218]. CTLA-4 is produced and mobilized from the internal side of the cell membrane, to the immune synapses 2 to 3 days after T-cell activation has taken place. There, it is bound to either one of the costimulatory molecules, CD80 and CD86. CTLA-4 expression turns the activated T cell to an inhibitory T cell. A delay in CTLA-4 expression favors T-cell activation and could be a pathway to improve or expand the immune response against tumors [218]. There are two CTLA-4-blocking antibodies for use in humans that have been mostly tested in patients with metastatic melanoma [235]. Recently, the fully human immunoglobulin G1 (IgG1) monoclonal antibody ipilimumab (Bristol-Myers Squibb, Princeton, NJ, USA) has demonstrated significant benefits in overall survival in randomized phase III studies in the first- or second-line treatment of metastatic melanoma [236, 237], gaining FDA approval. Clinical research of anti-CTLA-4 in hematologic tumors has been scarce to date. However, a phase 1 dose escalation trial with ipilimumab in the setting of allohematopoietic cell transplantation for relapsed hematologic malignancies reported interesting results [238] (Table 2). This trial tried to assess the efficacy of ipilimumab in augmenting the graft versus malignancy (GVM) effect. Among 14 patients with relapsed HL, 2 achieved a durable complete response and other 2 patients who had evidence of rapid progression achieved disease stabilization after ipilimumab [238]. This clinical effect in a highly pretreated population represents a proof of principle of activity of the anti-CTLA4 antibodies in HL and merits further investigation.

PD-1 is expressed on activated T and B cells, natural killer, dendritic cells, and activated monocytes [239]. PD-1 plays a major role in maintenance of T-cell tolerance limiting effector T-cell responses. There are two ligands of PD1, PD-L1, and PD-L2 (or B7-H1 and B7-H2) [240]. PD-L1 is aberrantly expressed in H/RS cells of Hodgkin's lymphoma and thus it can induce immune suppression through signaling PD-1 [33]. PD-L1-PD-1 signaling system is operative in patients with HL, and TILs around H/RS cells seem to be kept in balance by this inhibitory signaling. These findings suggest a plausible mechanism for deficient cellular immunity observed in HL patients and propose a potentially effective immunologic strategy for the treatment of HL.

CD40 is a member of the tumor necrosis factor receptor family expressed on macrophages, dendritic cells, endothelial and B cells, and fibroblasts [241]. Binding of CD40 with its CD40 ligand (CD40L) or CD154 acts on APC and T cells mediate both cellular and humoral responses. Specifically on APC, CD40 plays a central role in priming and expansion of antigen-specific CD4 T cells by regulating the expression of costimulatory molecules on APC such as CD80 and CD86 (B7.1 and B7.2) and by production of cytokines such as IL-12, IL-8, or TNF-*α* [242]. The functional role of CD40/CD40L and interferon regulatory factor 4 (IRF4) in Hodgkin's lymphoma microenvironment seems to be extremely important in HL [243]. A phase I study of the humanized anti-CD40 monoclonal antibody dacetuzumab in 50 patients with refractory or recurrent non-HL has been performed showing an acceptable safety profile and modest activity with 6 objective responses reported [244].

OX-40 is a member of the tumor necrosis factor (TNF) superfamily that needs T-cell activation to be expressed [259]. OX-40 is present in CD4^+^ and CD8^+^ T cells, whereas its ligand OX40L is expressed on activated APC, B cells, and macrophages [260]. Engagement of OX40L with the OX40 receptor is essential for the proliferation and survival of T cells leading to a larger expansion of effector T and antigen-specific memory T cells [260]. In addition, OX40 signaling increases cytokine secretion by CD4^+^T cells and enhances the development of Th1 and Th2 cells. Recently, it has been demonstrated that histone deacetylase inhibitors (HDACIs) may have a favorable antitumor effect by regulating the expression of OX40L in HL [261]. Clinical responses achieved in relapsed and heavily pretreated HL with some HDACIs like vorinostat, mocetinostat, or panobinostat might be mediated by the upregulation of OX40L in HL cells [262].

### 7.4. Monoclonal Antibodies Targeting HL Microenvironment

CD20 and CD52 molecules are not commonly expressed on the H/RS cells; however, the surrounding cells of the characteristic HL microenvironment commonly express extensively these antigens. Thus, interfering the crosstalk between H/RS cells and their cellular partners with monoclonal antibodies against CD20 and CD52 may represent an attractive therapeutic strategy to explore in clinical research.

The monoclonal antibody anti-CD20 rituximab is one of the therapeutic strategies aimed to deplete the HL microenvironment of normal B cells required for tumor cell growth. Specifically in classical HL, it has shown activity as single agent. In a pilot study, 5 out of 24 heavily pretreated patients with relapsed/refractory cHL treated with rituximab achieved a clinical response [247]. Interestingly, responses were achieved in patients with CD20-H/RS cells. Rituximab has also been tested combined with chemotherapies like ABVD and gemcitabine. Specifically with gemcitabine as salvage therapy it has demonstrated surprising and unexpected high overall response rates (48–88%) [248] (Table 2). Impact of rituximab on tumor microenvironment by depleting benign CD20^+^ cells is postulated as the main antineoplastic mechanism of action of this drug in HL, independently of CD20 expression on the RS cells. Reactive B-cell depletion in HL is being further tested by the use of anti-CD20 radio-immunoconjugates (90Y-ibritumomab tiuxetan and 131I-tositumomab) with pilot studies completed reporting favourable results in terms of tumour response and symptom control [263].

Alemtuzumab, a humanized IgG1*γ* monoclonal antibody directed against CD52, has shown notable activity as monotherapy for chronic lymphocytic leukemia [264]. Although its detailed mechanism of action is not completely clear, the binding of alemtuzumab to CD52 on target cells may cause cell death by three different mechanisms: complement-dependent cytotoxicity, antibody-dependent cellular cytotoxicity (ADCC), and apoptosis [250]. Since RS cells of HL do not express CD52, only surrounding cells such as neutrophils, eosinophils, macrophages, mast cells, and B and T cells, that strongly express the CD52 antigen, would be the targets for alemtuzumab and thus might be depleted by this antibody, depriving the RS cells of their critical survival factors. Although clinical trials are lacking in HL, results from a reduced-intensity conditioning allotransplantation study in relapsed HL suggest that alemtuzumab-induced elimination of infiltrating T cells may critically impact on the efficacy of the procedure and on the ability of donor lymphocytes in eradicating residual malignancy [251] (Table 2).

### 7.5. Lenalidomide

Other biological compounds with significant effects upon tumor microenvironment like lenalidomide are under clinical investigation, and at this moment represent one of the most promising therapeutical strategies in HL [253]. Lenalidomide (Revlimid), a thalidomide-derivate, belongs to a novel class of immunomodulatory drugs (IMIDs) approved for the treatment of multiple myeloma and myelodysplastic syndrome with deletion (-q5) [253]. Lenalidomide has multiple modes of action, including direct induction of apoptosis in tumour cells, antiangiogenic effects, and the activation of immune cells, such as natural killer cells and T cells, enhancing Th1-type cellular immunity and natural killer T-cell cytotoxicity [253]. Preliminary results of some clinical trials of lenalidomide in HL have been recently reported [254–256]. In all studies, administration of oral daily lenalidomide in heavily pretreated HL patients induced clinical response ranging from 17 to 50%, and in most other patients disease stabilization was achieved. This pattern of response appears fully compatible with the predicted actions of lenalidomide towards the HL microenvironment. Results of a serie of HL treated with lenalidomide were of great interest at this point [265]. 12 patients with relapsed or refractory HL were included in this program. All patients had relapsed after at least four chemotherapies, and, except two patients, all had previously undergone high dose chemotherapy and autologous stem cell transplantation. Most patients had not responded to the previous treatment. With respect to clinical outcome, none of the twelve patients showed radiological evidence of progression after two cycles of lenalidomide. Overall response rate was 50% (6 of 12), with 5 partial responses and 1 complete remission, in addition six patients had stable disease after two cycles [265] (Table 2).

### 7.6. Anti-CD30 Monoclonal Antibodies

The member of the tumor-necrosis-factor- (TNF-) receptor family CD30 is expressed abundantly on Reed-Sternberg cells of HL [266]. CD30 has pleiotropic biologic functions, being capable of promoting cell proliferation and survival as well as inducing antiproliferative responses and cell death. Final effects of CD30 activation seem largely dependent on the microenvironment context [266]. Unconjugated anti-CD30 antibodies have been tested in phase I and II studies showing limited clinical activity. On the contrary, the use of antibody-drug conjugates (ADCs) has rendered better results [267]. Brentuximab vedotin (SGN-35) is an ADC consisting of chimeric anti-CD30 antibody cAC10 (SGN-30) conjugated to the tubulin destabilizer monomethyl auristatin E (MMAE) [267]. In the first in human phase I dose escalation study, brentuximab vedotin was administered to 45 patients with relapsed or refractory CD30-positive lymphomas, primarily HL and anaplastic large cell lymphoma (ALCL) [257]. Brentuximab vedotin showed a good safety profile and objective response was observed in 17 (38%) patients, including 11 (24%) complete remissions. Tumor regression was observed in 86% of patients. Results of a phase II trial in relapsed HL has been recently communicated [258]. 102 patients were enrolled with a median age of 31 years and all were required to have failed an ASCT. Overall response was achieved in 76 of 102 patients (ORR: 75%) with 35 complete responses (CR: 34%). After these impressive results, brentuximab was recommended for an accelerated approval by the FDA and in August 2011, was approved in the US for the treatment of HL after failure of autologous stem cell transplant (ASCT) or after failure of at least two prior multiagent chemotherapy regimens in ASCT-ineligible candidates (Table 2).

## 8. Conclusions

The recent research activities led to a better understanding of the phenotype, molecular characteristics, histogenesis, and possible mechanisms of HL lymphomagenesis. New pathologic factors have been studied recently, showing that HL can be differentiated through its specific cellular microenvironment. The interplay between tumoral cells and the reactive microenvironment determines not only the histological morphology and classification but also the clinicopathological features of HL patients. Importantly, this may correlate also with the clinical course of disease and the final long-term outcomes. However, recent advances in our understanding of HL biology and immunology seem to indicate that infiltrated immune cells in the tumoral microenvironment may play different, even opposite, functions according to the signals it senses. It is critical to understand what happens in the tumoral microenvironment in order to design fine tune approaches that may modulate immune response toward cancer cell destruction. Strategies aimed at interfering with the crosstalk between H/RS cells and their cellular partners have been taken into account in the development of new immunotherapys that target different cell components of HL microenvironment. Combination strategies of chemotherapy, especially with anthracyclines and gemcitabine, radiotherapy, and immunotherapy will eventually synergize and obtain meaningful clinical results.

In our opinion there exist large amount of data which provides sufficient evidence to consider the host immune reaction as one of the main determinants of the clinical evolution in HL. Importantly, this immune response is capable of being modulated in clinic, so new therapeutical strategies based on combinatorial approaches with the ability of boosting immune responses might not be neglected in the coming future to improve the chance of cure of our patients with HL.

## Figures and Tables

**Table 1:** Recompilation of the different factors implicated in the tumoral immune escape in HL.

Strategy	Mechanisms	Regulated factors
Tumoral protective action	Upregulation of growth and survival receptors expression [6]	IL-7R, IL-9R, IL-13R, TACI, and CCR5 tumoral cells
	Downregulation of transcription factors [71]	IL-6R, TACI, RANK, TNFR-1, Cys-LT receptors, and NOTCH-1
	Upregulation of Th2 cells attractant chemokines [28]	TARC, MDC
	Upregulation of apoptosis/proliferation modulators [20, 28, 72–74]	Fas, FasL, IL-1*β*, TGF-*β*, TNFR, IL-13, IL-3
	Upregulation of immunoregulatory protein and regulatory T cells [27, 33]	Gal-1, PD1
	Downregulation of adhesion factors [79]	HGF, c-MET
	Downregulation of cytotoxic cells activity [75, 77, 126–135]	MHC I, PI9, IL-10, TGF-*β*, LAG-3, CTLA-4
	Upregulation of inhibitory T cells activator [31]	PGE2
	Selection of minor side population [87, 88, 91]	MDR1, ABCG2, gemcitabine resistance factor

Reprogramming of tumoral cells	Mutations/downregulation of MHC class II [92]	CIITA
	Upregulation of death receptors ligands [33]	PDL1
	Upregulation of immunosuppressive factors [32, 93]	TGF-*β*, PD-1

Tolerance induction by TAM	Macrophage deviation to Th2 differentiation (TAM) [110, 112, 113]	IL-6, TNF, IL-1*β*, IL-23
	Upregulation of inflammatory and matrix-remodeling genes [125]	C1Qalpha, C1Qbeta, and CXCL9

Tolerance induction by Tregs	Conversion of naïve regulatory T cells to CD4^+^ CD25^+^ [101, 102]	Foxp3
	Dowregulation of CTL activity [104, 105]	IL-2R*α* (CD25), Il-10, TNF-*β*

**Table 2:** Clinical experience with new immunotherapies in Hodgkin lymphoma.

Agent	Mechanism of action	Clinical development status	References
Ipilimumab	Anti-CTL4 Mo Ab	Phase I	[245]
Rituximab	Anti-CD20 Mo Ab	Pilot studies	[246–248]
		Phase II (combined with CT)	
90Y-ibritumomab tiuxetan	Anti-CD20	Pilot studies	[249]
	radio-immunoconjugate		
Alemtuzumab	Anti-CD52 Mo Ab	Pilot-phase II studies	[250, 251]
Lenalidomide	Immunomodulatory	Phase II	[252–256]
Brentuximab vedotin	Antibody-drug conjugate	Phase II	[ 257, 258]
	(anti-CD30 plus tubulin destabilizer)		

Mo Ab: monoclonal antibody. CT: chemotherapy.

## References

[1] Küppers R (2002). Molecular biology of Hodgkin’s lymphoma. *Advances in Cancer Research*.

[2] Staudt LM (2000). The molecular and cellular origins of Hodgkin’s disease. *Journal of Experimental Medicine*.

[3] Thomas RK, Re D, Wolf J, Diehl V (2004). Part I: Hodgkin’s lymphoma—molecular biology of Hodgkin and Reed-Sternberg cells. *Lancet Oncology*.

[4] Swerdlow SH, Campo E, Harris NL (2008). *WHO Classification of Tumours of Haematopoietic and Lymphoid Tissues*.

[5] Swinnen LJ (2001). Post-transplant lymphoproliferative disorders: implications for acquired immunodeficiency syndrome-associated malignancies. *Journal of the National Cancer Institute. Monographs*.

[6] Aldinucci D, Gloghini A, Pinto A, De Filippi R, Carbone A (2010). The classical Hodgkin’s lymphoma microenvironment and its role in promoting tumour growth and immune escape. *Journal of Pathology*.

[7] Poppema S (2005). Immunobiology and pathophysiology of Hodgkin lymphomas. *Hematology*.

[8] Machado L, Jarrett R, Morgan S (2009). Expression and function of T cell homing molecules in Hodgkin’s lymphoma. *Cancer Immunology, Immunotherapy*.

[9] Poppema S, Potters M, Visser L, van den Berg AM (1998). Immune escape mechanisms in Hodgkin’s disease. *Annals of Oncology*.

[10] Álvaro-Naranjo T, Lejeune M, Salvadó-Usach MT (2005). Tumor-infiltrating cells as a prognostic factor in Hodgkin’s lymphoma: a quantitative tissue microarray study in a large retrospective cohort of 267 patients. *Leukemia and Lymphoma*.

[11] Oudejans JJ, Jiwa NM, Kummer JA (1997). Activated cytotoxic T cells as prognostic marker in Hodgkin’s disease. *Blood*.

[12] Stein H, Delsol G, Pileri S (2008). Classical Hodgkin lymphoma, introduction. *WHO Classification of Tumours of Haematopoietic and Lymphoid Tissues*.

[13] Poppema S (2008). Nodular lymphocyte predominant Hodgkin lymphoma. *WHO Classification of Tumors of the Hematopoietic and Lymphoid Tissue*.

[14] Stein R, Von Wasielewski R, Poppema S, MacLennan K, Guenova M (2008). Nodular sclerosis classical Hodgkin lymphoma. *WHO Classification of Tumors of Haematopoietic and Lymphoid Tissues IARC*.

[15] Weiss L, Von Wasielewski R, Delsol G, Poppema S, Stein H (2008). Mixed cellularity classical Hodgkin lymphoma. *WHO Classification of Tumors of the Hematopoietic and Lymphoid Tissue*.

[16] Anagnostopoulos I, Isaacson P, Stein H (2008). Lymphocyte rich classical Hodgkin lymphoma. *WHO Classification of Tumors of the Hematopoietic and Lymphoid Tissue*.

[17] Benharroch D, Stein H, Peh S (2008). Lymphocyte-depleted classical Hodgkin lymphoma. *WHO Classification of Tumors of the Hematopoietic and Lymphoid Tissue*.

[18] Kapp U, Dux A, Schell-Frederick E (1994). Disseminated growth of Hodgkin’s-derived cell lines L540 and L540cy in immune-deficient SCID mice. *Annals of Oncology*.

[19] Biggar RJ, Jaffe ES, Goedert JJ, Chaturvedi A, Pfeiffer R, Engels EA (2006). Hodgkin lymphoma and immunodeficiency in persons with HIV/AIDS. *Blood*.

[20] Aldinucci D, Olivo K, Lorenzon D (2005). The role of interleukin-3 in classical Hodgkin’s disease. *Leukemia and Lymphoma*.

[21] Aldinucci D, Lorenzon D, Olivo K, Rapanà B, Gattei V (2004). Interactions between tissue fibroblasts in lymph nodes and Hodgkin/Reed-Sternberg cells. *Leukemia and Lymphoma*.

[22] Jundt F, Anagnostopoulos I, Bommert K (1999). Hodgkin/Reed-Sternberg cells induce fibroblasts to secrete eotaxin, a potent chemoattractant for T cells and eosinophils. *Blood*.

[23] Tanijiri T, Shimizu T, Uehira K (2007). Hodgkin’s Reed-Sternberg cell line (KM-H2) promotes a bidirectional differentiation of CD4^+^CD25^+^Foxp3^+^ T cells and CD4^+^ cytotoxic T lymphocytes from CD4^+^ naive T cells. *Journal of Leukocyte Biology*.

[24] Álvaro T, Lejeune M, Salvadó MT (2005). Outcome in Hodgkin’s lymphoma can be predicted from the presence of accompanying cytotoxic and regulatory T cells. *Clinical Cancer Research*.

[25] Kelley TW, Pohlman B, Elson P, Hsi ED (2007). The ratio of FOXP3^+^ regulatory T cells to granzyme B^+^ cytotoxic T/NK cells predicts prognosis in classical Hodgkin lymphoma and is independent of bcl-2 and MAL expression. *American Journal of Clinical Pathology*.

[26] Tan TT, Coussens LM (2007). Humoral immunity, inflammation and cancer. *Current Opinion in Immunology*.

[27] Juszczynski P, Ouyang J, Monti S (2007). The AP1-dependent secretion of galectin-1 by Reed-Sternberg cells fosters immune privilege in classical Hodgkin lymphoma. *Proceedings of the National Academy of Sciences of the United States of America*.

[28] Skinnider BF, Mak TW (2002). The role of cytokines in classical Hodgkin lymphoma. *Blood*.

[29] Gandhi MK, Moll G, Smith C (2007). Galectin-1 mediated suppression of Epstein-Barr virus-specific T-cell immunity in classic Hodgkin lymphoma. *Blood*.

[30] Newcom SR, Gu L (1995). Transforming growth factor *β*1 messenger RNA in Reed-Sternberg cells in nodular sclerosing Hodgkin’s disease. *Journal of Clinical Pathology*.

[31] Chemnitz JM, Driesen J, Classen S (2006). Prostaglandin E2 impairs CD4^+^ T cell activation by inhibition of lck: implications in Hodgkin’s lymphoma. *Cancer Research*.

[32] Chemnitz JM, Eggle D, Driesen J (2007). RNA fingerprints provide direct evidence for the inhibitory role of TGF*β* and PD-1 on CD4^+^ T cells in Hodgkin lymphoma. *Blood*.

[33] Yamamoto R, Nishikori M, Kitawaki T (2008). PD-1 PD-1 ligand interaction contributes to immunosuppressive microenvironment of Hodgkin lymphoma. *Blood*.

[34] Álvaro T, de la Cruz-Merino L, Henao-Carrasco F (2010). Tumor microenvironment and immune effects of antineoplastic therapy in lymphoproliferative syndromes. *Journal of Biomedicine and Biotechnology*.

[35] Noguera R, Nieto OA, Tadeo I, Farinas F, Alvaro T (2012). Extracellular matrix, biotensegrity and tumor microenvironment. An update and overview. *Histology and Histopathology*.

[36] Cabannes E, Khan G, Aillet F, Jarrett RF, Hay RT (1999). Mutations in the IkBa gene in Hodgkin’s disease suggest a tumour suppressor role for I*κ*B*α*. *Oncogene*.

[37] Krappmann D, Emmerich F, Kordes U, Scharschmidt E, Dörken B, Scheidereit C (1999). Molecular mechanisms of constitutive NF-*κ*B/Rel activation in Hodgkin/Reed-Sternberg cells. *Oncogene*.

[38] Jungnickel B, Staratschek-Jox A, Bräuninger A (2000). Clonal deleterious mutations in the i*κ*b*α* gene in the malignant cells in Hodgkin’s lymphoma. *Journal of Experimental Medicine*.

[39] Emmerich F, Theurich S, Hummel M (2003). Inactivating I kappa B epsilon mutations in Hodgkin/Reed-Sternberg cells. *Journal of Pathology*.

[40] Emmerich F, Meiser M, Hummel M (1999). Overexpression of I kappa B alpha without inhibition of NF-*κ*B activity and mutations in the I kappa B alpha gene in Reed-Sternberg cells. *Blood*.

[41] Takacs M, Segesdi J, Banati F (2009). The importance of epigenetic alterations in the development of epstein-barr virus-related lymphomas. *Mediterranean Journal of Hematology and Infectious Diseases*.

[42] Carbone A, Spina M, Gloghini A, Tirelli U (2011). Classical Hodgkin’s lymphoma arising in different host’s conditions: pathobiology parameters, therapeutic options, and outcome. *American Journal of Hematology*.

[43] Serraino D, Boschini A, Carrieri P (2000). Cancer risk among men with, or at risk of, HIV infection in southern Europe. *AIDS*.

[44] Franceschi S, Dal Maso L, Pezzotti P (2003). Incidence of AIDS-defining cancers after AIDS diagnosis among people with AIDS in Italy, 1986–1998. *Journal of Acquired Immune Deficiency Syndromes*.

[45] Clifford GM, Rickenbach M, Lise M (2009). Hodgkin lymphoma in the Swiss HIV Cohort Study. *Blood*.

[46] Grogg KL, Miller RF, Dogan A (2007). HIV infection and lymphoma. *Journal of Clinical Pathology*.

[47] Carbone A, Gloghini A, Serraino D, Spina M (2009). HIV-associated Hodgkin lymphoma. *Current Opinion in HIV and AIDS*.

[48] Tirelli U, Errante D, Dolcetti R (1995). Hodgkin’s disease and human immunodeficiency virus infection: clinicopathologic and virologic features of 114 patients from the Italian Cooperative Group on AIDS and Tumors. *Journal of Clinical Oncology*.

[49] Vaccher E, Spina M, Talamini R (2003). Improvement of systemic human immunodeficiency virus-related non-hodgkin lymphoma outcome in the era of highly active antiretroviral therapy. *Clinical Infectious Diseases*.

[50] Chimienti E, Spina M, Gastaldi R (2008). Clinical characteristics and outcome of 290 patients (pts) with Hodgkin’s disease and HIV infection (HD-HIV) in pre and HAART (highly active antiretroviral therapy) era. *Annals of Oncology*.

[51] Krishnan A, Molina A, Zaia J (2005). Durable remissions with autologous stem cell transplantation for high-risk HTV-associated lymphomas. *Blood*.

[52] Serrano D, Carrión R, Balsalobre P (2005). HIV-associated lymphoma successfully treated with peripheral blood stem cell transplantation. *Experimental Hematology*.

[53] Re A, Michieli M, Casari S (2009). High-dose therapy and autologous peripheral blood stem cell transplantation as salvage treatment for AIDS-related lymphoma: long-term results of the Italian Cooperative Group on AIDS and Tumors (GICAT) study with analysis of prognostic factors. *Blood*.

[54] Hayashi RJ, Kraus MD, Patel AL (2001). Posttransplant lymphoproliferative disease in children: correlation of histology to clinical behavior. *Journal of Pediatric Hematology/Oncology*.

[55] Midthun DE, Mcüougall JC, Peters SG, Scott JP (1997). Medical management and complications in the lung transplant recipient. *Mayo Clinic Proceedings*.

[56] Swerdlow S, Campo E, Harris N Pathology and genetics of tumours of haematopoietic and lymphoid tissues.

[57] Rohr JC, Wagner HJ, Lauten M (2008). Differentiation of EBV-induced post-transplant Hodgkin lymphoma from Hodgkin-like post-transplant lymphoproliferative disease. *Pediatric Transplantation*.

[58] Knight JS, Tsodikov A, Cibrik DM, Ross CW, Kaminski MS, Blayney DW (2009). Lymphoma after solid organ transplantation: risk, response to therapy, and survival at a transplantation center. *Journal of Clinical Oncology*.

[59] Adams H, Campidelli C, Dirnhofer S, Pileri SA, Tzankov A (2009). Clinical, phenotypic and genetic similarities and disparities between post-transplant and classical Hodgkin lymphomas with respect to therapeutic targets. *Expert Opinion on Therapeutic Targets*.

[60] Pham PTT, Wilkinson AH, Gritsch HA (2002). Monotherapy with the anti-CD20 monoclonal antibody rituximab in a kidney transplant recipient with posttransplant lymphoproliferative disease. *Transplantation Proceedings*.

[61] Diehl V, Stein H, Hummel M, Zollinger R, Connors JM (2003). Hodgkin’s lymphoma: biology and treatment strategies for primary, refractory, and relapsed disease. *Hematology*.

[62] Josting A, Nogová L, Franklin J (2005). Salvage radiotherapy in patients with relapsed and refractory Hodgkin’s lymphoma: a retrospective analysis from the German Hodgkin Lymphoma Study Group. *Journal of Clinical Oncology*.

[63] Tzankov A, Krugmann J, Fend F, Fischhofer M, Greil R, Dirnhofer S (2003). Prognostic significance of CD20 expression in classical Hodgkin lymphoma: a clinicopathological study of 119 cases. *Clinical Cancer Research*.

[64] Donnelly G, Filippa D, Moskowitz C (1999). Increased treatment failure in patients with CD20 positive classic Hodgkin disease (HD). *Blood*.

[65] Rassidakis GZ, Medeiros LJ, Viviani S (2002). CD20 expression in Hodgkin and Reed-Sternberg cells of classical Hodgkin’s disease: associations with presenting features and clinical outcome. *Journal of Clinical Oncology*.

[66] Portlock CS, Donnelly GB, Qin J (2004). Adverse prognostic significance of CD20 positive Reed-Sternberg cells in classical Hodgkin’s disease. *British Journal of Haematology*.

[67] Canioni D, Deau-Fischer B, Taupin P (2009). Prognostic significance of new immunohistochemical markers in refractory classical Hodgkin lymphoma: a study of 59 cases. *PLoS ONE*.

[68] Linch DC, Winfield D, Goldstone AH (1993). Dose intensification with autologous bone-marrow transplantation in relapsed and resistant Hodgkin’s disease: results of a BNLI randomised trial. *The Lancet*.

[69] Schmitz N, Pfistner B, Sextro M (2002). Aggressive conventional chemotherapy compared with high-dose chemotherapy with autologous haemopoietic stem-cell transplantation for relapsed chemosensitive Hodgkin’s disease: a randomised trial. *The Lancet*.

[70] Fozza C, Longinotti M (2011). T-cell traffic jam in Hodgkin’s lymphoma: pathogenetic and therapeutic implications. *Advances in Hematology*.

[71] Küppers R (2009). Molecular biology of Hodgkin lymphoma. *Hematology*.

[72] Atkinson EA, Bleackley RC (1995). Mechanisms of lysis by cytotoxic T cells. *Critical Reviews in Immunology*.

[73] Berke G (1995). The CTL’s kiss of death. *Cell*.

[74] de Visser KE, Kast WM (1999). Effects of TGF-*β* on the immune system: implications for cancer immunotherapy. *Leukemia*.

[75] Bladergroen BA, Meijer CJLM, Ten Berge RL (2002). Expression of the granzyme B inhibitor, protease inhibitor 9, by tumor cells in patients with non-Hodgkin and Hodgkin lymphoma: a novel protective mechanism for tumor cells to circumvent the immune system?. *Blood*.

[76] Poppema S, Potters M, Emmens R, Visser L, van den Berg A (1999). Immune reactions in classical Hodgkin’s lymphoma. *Seminars in Hematology*.

[77] Kosmaczewska A, Frydecka I, Boćko D, Ciszak L, Teodorowska R (2002). Correlation of blood lymphocyte CTLA-4 (CD152) induction in Hodgkin’s disease with proliferative activity, interleukin 2 and interferon-gamma production. *British Journal of Haematology*.

[78] Su CC, Chiu HH, Chang CC, Chen JC, Hsu SM (2004). CD30 Is Involved in Inhibition of T-Cell Proliferation by Hodgkin’s Reed-Sternberg Cells. *Cancer Research*.

[79] Teofili L, Di Febo AL, Pierconti F (2001). Expression of the c-met proto-oncogene and its ligand, hepatocyte growth factor, in Hodgkin disease. *Blood*.

[80] Reis L, Melbert D, Krapcho M (2008). *SEER Cancer Statistics Review, 1975–2005*.

[81] Goodell MA, Brose K, Paradis G, Conner AS, Mulligan RC (1996). Isolation and functional properties of murine hematopoietic stem cells that are replicating in vivo. *Journal of Experimental Medicine*.

[82] Goodell MA, Rosenzweig M, Kim H (1997). Dye efflux studies suggest that hematopoietic stem cells expressing low or undetectable levels of CD34 antigen exist in multiple species. *Nature Medicine*.

[83] Hirschmann-Jax C, Foster AE, Wulf GG (2004). A distinct “side population” of cells with high drug efflux capacity in human tumor cells. *Proceedings of the National Academy of Sciences of the United States of America*.

[84] Patrawala L, Calhoun T, Schneider-Broussard R, Zhou J, Claypool K, Tang DG (2005). Side population is enriched in tumorigenic, stem-like cancer cells, whereas ABCG2^+^ and ABCG2^−^ cancer cells are similarly tumorigenic. *Cancer Research*.

[85] Moshaver B, Van Rhenen A, Kelder A (2008). Identification of a small subpopulation of candidate leukemia-initiating cells in the side population of patients with acute myeloid leukemia. *Stem Cells*.

[86] Ho MM, Ng AV, Lam S, Hung JY (2007). Side population in human lung cancer cell lines and tumors is enriched with stem-like cancer cells. *Cancer Research*.

[87] Bunting KD (2002). ABC transporters as phenotypic markers and functional regulators of stem cells. *Stem Cells*.

[88] Zhou S, Schuetz JD, Bunting KD (2001). The ABC transporter Bcrp1/ABCG2 is expressed in a wide variety of stem cells and is a molecular determinant of the side-population phenotype. *Nature Medicine*.

[89] Austin Doyle L, Yang W, Abruzzo LV (1998). A multidrug resistance transporter from human MCF-7 breast cancer cells. *Proceedings of the National Academy of Sciences of the United States of America*.

[90] Plasschaert SLA, de Bont ESJM, Boezen M (2005). Expression of multidrug resistance-associated proteins predicts prognosis in childhood and adult acute lymphoblastic leukemia. *Clinical Cancer Research*.

[91] Shafer JA, Cruz CR, Leen AM (2010). Antigen-specific cytotoxic T lymphocytes can target chemoresistant side-population tumor cells in Hodgkin lymphoma. *Leukemia and Lymphoma*.

[92] Steidl C, Shah SP, Woolcock BW (2011). MHC class II transactivator CIITA is a recurrent gene fusion partner in lymphoid cancers. *Nature*.

[93] Muenst S, Hoeller S, Dirnhofer S, Tzankov A (2009). Increased programmed death-1+ tumor-infiltrating lymphocytes in classical Hodgkin lymphoma substantiate reduced overall survival. *Human Pathology*.

[94] Curiel TJ, Coukos G, Zou L (2004). Specific recruitment of regulatory T cells in ovarian carcinoma fosters immune privilege and predicts reduced survival. *Nature Medicine*.

[95] Fontenot JD, Gavin MA, Rudensky AY (2003). Foxp3 programs the development and function of CD4^+^CD25^+^ regulatory T cells. *Nature Immunology*.

[96] Suri-Payer E, Amar AZ, Thornton AM, Shevach EM (1998). CD4^+^CD25^+^ T cells inhibit both the induction and effector function of autoreactive T cells and represent a unique lineage of immunoregulatory cells. *Journal of Immunology*.

[97] Sakaguchi S, Miyara M, Costantino CM, Hafler DA (2010). FOXP3^+^ regulatory T cells in the human immune system. *Nature Reviews Immunology*.

[98] Shimizu J, Yamazaki S, Takahashi T, Ishida Y, Sakaguchi S (2002). Stimulation of CD25^+^CD4^+^ regulatory T cells through GITR breaks immunological self-tolerance. *Nature Immunology*.

[99] Sutmuller RPM, van Duivenvoorde LM, van Elsas A (2001). Synergism of cytotoxic T lymphocyte-associated antigen 4 blockade and depletion of CD25^+^ regulatory T cells in antitumor therapy reveals alternative pathways for suppression of autoreactive cytotoxic T lymphocyte responses. *Journal of Experimental Medicine*.

[100] Takahashi T, Tagami T, Yamazaki S (2000). Immunologic self-tolerance maintained by CD25^+^CD4^+^ regulatory T cells constitutively expressing cytotoxic T lymphocyte-associated antigen 4. *Journal of Experimental Medicine*.

[101] Karube K, Ohshima K, Tsuchiya T (2004). Expression of FoxP3, a key molecule in CD4^+^CD25^+^ regulatory T cells, in adult T-cell leukaemia/lymphoma cells. *British Journal of Haematology*.

[102] Hori S, Nomura T, Sakaguchi S (2003). Control of regulatory T cell development by the transcription factor Foxp3. *Science*.

[103] Teichmann M, Meyer B, Beck A, Niedobitek G (2005). Expression of the interferon-inducible chemokine IP-10 (CXCL10), a chemokine with proposed anti-neoplastic functions, in Hodgkin lymphoma and nasopharyngeal carcinoma. *Journal of Pathology*.

[104] Azuma T, Takahashi T, Kunisato A, Kitamura T, Hirai H (2003). Human CD4^+^ CD25^+^ regulatory T cells suppress NKT cell functions. *Cancer Research*.

[105] Wolf AM, Wolf D, Steurer M, Gastl G, Gunsilius E, Grubeck-Loebenstein B (2003). Increase of regulatory T cells in the peripheral blood of cancer patients. *Clinical Cancer Research*.

[106] Yamaguchi T, Sakaguchi S (2006). Regulatory T cells in immune surveillance and treatment of cancer. *Seminars in Cancer Biology*.

[107] Wang HY, Wang RF (2007). Regulatory T cells and cancer. *Current Opinion in Immunology*.

[108] Ostrand-Rosenberg S, Sinha P (2009). Myeloid-derived suppressor cells: linking inflammation and cancer. *Journal of Immunology*.

[109] Movahedi K, Laoui D, Gysemans C (2010). Different tumor microenvironments contain functionally distinct subsets of macrophages derived from Ly6C(high) monocytes. *Cancer Research*.

[110] Zamarron BF, Chen W (2011). Dual roles of immune cells and their factors in cancer development and progression. *International Journal of Biological Sciences*.

[111] Biswas SK, Mantovani A (2010). Macrophage plasticity and interaction with lymphocyte subsets: cancer as a paradigm. *Nature Immunology*.

[112] Khramtsova G, Liao C, Khramtsov A (2009). The M2/alternatively activated macrophage phenotype correlates with aggressive histopathologic features and poor clinical outcome in early stage breast cancer. *Cancer Research*.

[113] Mantovani A, Sozzani S, Locati M, Allavena P, Sica A (2002). Macrophage polarization: tumor-associated macrophages as a paradigm for polarized M2 mononuclear phagocytes. *Trends in Immunology*.

[114] Pollard JW (2004). Tumour-educated macrophages promote tumour progression and metastasis. *Nature Reviews Cancer*.

[115] Talmadge JE, Donkor M, Scholar E (2007). Inflammatory cell infiltration of tumors: jekyll or Hyde. *Cancer and Metastasis Reviews*.

[116] Bingle L, Brown NJ, Lewis CE (2002). The role of tumour-associated macrophages in tumour progression: implications for new anticancer therapies. *Journal of Pathology*.

[117] Condeelis J, Pollard JW (2006). Macrophages: obligate partners for tumor cell migration, invasion, and metastasis. *Cell*.

[118] Mantovani A, Allavena P, Sica A (2004). Tumour-associated macrophages as a prototypic type II polarised phagocyte population: role in tumour progression. *European Journal of Cancer*.

[119] Pollard JW (2008). Macrophages define the invasive microenvironment in breast cancer. *Journal of Leukocyte Biology*.

[120] Clark CE, Hingorani SR, Mick R, Combs C, Tuveson DA, Vonderheide RH (2007). Dynamics of the immune reaction to pancreatic cancer from inception to invasion. *Cancer Research*.

[121] Dave SS, Wright G, Tan B (2004). Prediction of survival in follicular lymphoma based on molecular features of tumor-infiltrating immune cells. *The New England Journal of Medicine*.

[122] Paik S, Shak S, Tang G (2004). A multigene assay to predict recurrence of tamoxifen-treated, node-negative breast cancer. *The New England Journal of Medicine*.

[123] Taskinen M, Karjalainen-Lindsberg ML, Nyman H, Eerola LM, Leppä S (2007). A high tumor-associated macrophage content predicts favorable outcome in follicular lymphoma patients treated with rituximab and cyclophosphamide- doxorubicin-vincristine-prednisone. *Clinical Cancer Research*.

[124] Steidl C, Lee T, Shah SP (2010). Tumor-associated macrophages and survival in classic Hodgkin’s lymphoma. *The New England Journal of Medicine*.

[125] Birgersdotter A, Baumforth KRN, Porwit A (2009). Inflammation and tissue repair markers distinguish the nodular sclerosis and mixed cellularity subtypes of classical Hodgkin’s lymphoma. *British Journal of Cancer*.

[126] Poppema S, Visser L (1994). Absence of HLA class I expression by Reed-Sternberg cells. *American Journal of Pathology*.

[127] Newcom SR, Kadin ME, Ansari AA, Diehl V (1988). L-428 nodular sclerosing Hodgkin’s cell secretes a unique transforming growth factor-beta active at physiologic pH. *Journal of Clinical Investigation*.

[128] Ohshima K, Suzumiya J, Akamatu M, Takeshita M, Kikuchi M (1995). Human and viral interleukin-10 in Hodgkin’s disease, and its influence on CD4^+^ and CD8^+^ T lymphocytes. *International Journal of Cancer*.

[129] Medema JP, de Jong J, Peltenburg LTC (2001). Blockade of the granzyme B/perforin pathway through overexpression of the serine protease inhibitor PI-9/SPI-6 constitutes a mechanism for immune escape by tumors. *Proceedings of the National Academy of Sciences of the United States of America*.

[130] Gandhi MK, Lambley E, Duraiswamy J (2006). Expression of LAG-3 by tumor-infiltrating lymphocytes is coincident with the suppression of latent membrane antigen-specific CD8^+^ T-cell function in Hodgkin lymphoma patients. *Blood*.

[131] Marshall NA, Vickers MA, Barker RN (2003). Regulatory T cells secreting IL-10 dominate the immune response to EBV latent membrane protein 1. *Journal of Immunology*.

[132] Suvas S, Kumaraguru U, Pack CD, Lee S, Rouse BT (2003). CD4^+^CD25^+^ T cells regulate virus-specific primary and memory CD8^+^ T cell responses. *Journal of Experimental Medicine*.

[133] Huard B, Mastrangeli R, Prigent P (1997). Characterization of the major histocompatibility complex class II binding site on LAG-3 protein. *Proceedings of the National Academy of Sciences of the United States of America*.

[134] Hannier S, Tournier M, Bismuth G, Triebel F (1998). CD3/TCR complex-associated lymphocyte activation gene-3 molecules inhibit CD3/TCR signaling. *Journal of Immunology*.

[135] Maçon-Lemaître L, Triebel F (2005). The negative regulatory function of the lymphocyte-activation gene-3 co-receptor (CD223) on human T cells. *Immunology*.

[136] Meng Y, Beckett MA, Liang H (2010). Blockade of tumor necrosis factor *α* signaling in tumor-associated macrophages as a radiosensitizing strategy. *Cancer Research*.

[137] Ahn GO, Tseng D, Liao CH, Dorie MJ, Czechowicz A, Brown JM (2010). Inhibition of Mac-1 (CD11b/CD18) enhances tumor response to radiation by reducing myeloid cell recruitment. *Proceedings of the National Academy of Sciences of the United States of America*.

[138] Molin D, Fischer M, Xiang Z (2001). Mast cells express functional CD30 ligand and are the predominant CD30L-positive cells in Hodgkin’s disease. *British Journal of Haematology*.

[139] Pagès F, Galon J, Dieu-Nosjean MC, Tartour E, Sautès-Fridman C, Fridman WH (2010). Immune infiltration in human tumors: a prognostic factor that should not be ignored. *Oncogene*.

[140] Bollard CM, Aguilar L, Straathof KC (2004). Cytotoxic T lymphocyte therapy for epstein-barr virus^+^ Hodgkin’s disease. *Journal of Experimental Medicine*.

[141] Alvaro T, Lejeune M, Escriva P (2009). Appraisal of immune response in lymphoproliferative syndromes: a systematic review. *Critical Reviews in Oncology/Hematology*.

[142] Diehl V, Thomas RK, Re D (2004). Part II: Hodgkin’s lymphoma—diagnosis and treatment. *Lancet Oncology*.

[143] Connors JM (2005). State-of-the-art therapeutics: Hodgkin’s lymphoma. *Journal of Clinical Oncology*.

[144] Specht L, Hasenclever D (1999). Prognostic factors of Hodgkin's disease. *Hodgkin's Disease*.

[145] Engert A, Plutschow A, Eich HT (2010). Reduced treatment intensity in patients with early-stage Hodgkin's lymphoma. *The New England Journal of Medicine*.

[146] Hasenclever D, Diehl V (1998). A prognostic score for advanced Hodgkin’s disease. *The New England Journal of Medicine*.

[147] Ansell SM, Stenson M, Habermann TM, Jelinek DF, Witzig TE (2001). CD4^+^ T-cell immune response to large B-cell non-Hodgkin’s lymphoma predicts patient outcome. *Journal of Clinical Oncology*.

[148] Willenbrock K, Roers A, Blohbaum B, Rajewsky K, Hansmann ML (2000). CD8^+^ T cells in Hodgkin’s disease tumor tissue are a polyclonal population with limited clonal expansion but little evidence of selection by antigen. *American Journal of Pathology*.

[149] Sanchez-Espiridion B, Montalban C, Lopez A (2010). A molecular risk score based on 4 functional pathways for advanced classical Hodgkin lymphoma. *Blood*.

[150] Bosshart H, Ansell SM, Jelinek DF, Wettstein PJ, Witzig TE (2002). T helper cell activation in B-cell lymphomas. *Journal of Clinical Oncology*.

[151] Kanavaros P, Vlychou M, Stefanaki K (1999). Cytotoxic protein expression in non-Hodgkin’s lymphomas and Hodgkin’s disease. *Anticancer Research A*.

[152] ten Berge RL, Oudejans JJ, Dukers DF, Meijer JWR, Ossenkoppele GJ, Meijer CJLM (2001). Percentage of activated cytotoxic T-lymphocytes in anaplastic large cell lymphoma and Hodgkin’s disease: an independent biological prognostic marker. *Leukemia*.

[153] Camilleri-Broët S, Fermé C, Berger F (2004). TiA1 in advanced-stage classical Hodgkin’s lymphoma: no prognostic impact for positive tumour cells or number of cytotoxic cells. *Virchows Archiv*.

[154] Kamper P, Bendix K, Hamilton-Dutoit S, Honoré B, Nyengaard JR, D’Amore F (2011). Tumor-infiltrating macrophages correlate with adverse prognosis and Epstein-Barr virus status in classical Hodgkin’s lymphoma. *Haematologica*.

[155] Leoncini L, Spina D, Megha T (1997). Cell kinetics, morphology, and molecular IgV(H) gene rearrangements in Hodgkin’s disease. *Leukemia and Lymphoma*.

[156] Bargou RC, Emmerich F, Krappmann D (1997). Constitutive nuclear factor-*κ*b-RelA activation is required for proliferation and survival of Hodgkin’s disease tumor cells. *Journal of Clinical Investigation*.

[157] Hinz M, Lemke P, Anagnostopoulos I (2002). Nuclear factor *κ*b-dependent gene expression profiling of Hodgkin’s disease tumor cells, pathogenetic significance, and link to constitutive signal transducer and activator of transcription 5a activity. *Journal of Experimental Medicine*.

[158] Hinz M, Löser P, Mathas S, Krappmann D, Dörken B, Scheidereit C (2001). Constitutive NF-*κ*B maintains high expression of a characteristic gene network, including CD40, CD86, and a set of antiapoptotic genes in Hodgkin/Reed-Sternberg cells. *Blood*.

[159] Izban KF, Ergin M, Huang Q (2001). Characterization of NF-*κ*B expression in Hodgkin’s disease: inhibition of constitutively expressed NF-*κ*B results in spontaneous caspase-independent apoptosis in Hodgkin and Reed-Sternberg cells. *Modern Pathology*.

[160] Mathas S, Hinz M, Anagnostopoulos I (2002). Aberrantly expressed c-Jun and JunB are a hallmark of Hodgkin lymphoma cells, stimulate proliferation and synergize with NF-*κ*B. *The EMBO Journal*.

[161] Sanchez-Beato M, Piris MA, Martinez-Montero JC (1996). MDM2 and p21WAF1/CIP1, wild-type p53-induced proteins, are regularly expressed by Sternberg-Reed cells in Hodgkin's disease. *The Journal of Pathology*.

[162] Küpper M, Joos S, von Bonin F (2001). MDM2 gene amplification and lack of p53 point mutations in Hodgkin and Reed-Sternberg cells: results from single-cell polymerase chain reaction and molecular cytogenetic studies. *British Journal of Haematology*.

[163] Morente MM, Piris MA, Abraira V (1997). Adverse clinical outcome in Hodgkin’s disease is associated with loss of retinoblastoma protein expression, high Ki67 proliferation index, and absence of Epstein-Barr Virus-latent membrane protein 1 expression. *Blood*.

[164] Montesinos-Rongen M, Roers A, Küppers R, Rajewsky K, Hansmann ML (1999). Mutation of the p53 gene is not a typical feature of Hodgkin and Reed- Sternberg cells in Hodgkin’s disease. *Blood*.

[165] García JF, Villuendas R, Algara P (1999). Loss of p16 protein expression associated with methylation of the p16(INK4A) gene is a frequent finding in Hodgkin’s disease. *Laboratory Investigation*.

[166] Montalbán C, García JF, Abraira V (2004). Influence of biologic markers on the outcome of Hodgkin’s lymphoma: a study by the Spanish Hodgkin’s Lymphoma Study Group. *Journal of Clinical Oncology*.

[167] Rassidakis GZ, Medeiros LJ, McDonnell TJ (2002). BAX expression in Hodgkin and Reed-Sternberg cells of Hodgkin’s disease: correlation with clinical outcome. *Clinical Cancer Research*.

[168] Rassidakis GZ, Medeiros LJ, Vassilakopoulos TP (2002). BCL-2 expression in Hodgkin and Reed-Sternberg cells of classical Hodgkin disease predicts a poorer prognosis in patients treated with ABVD or equivalent regimens. *Blood*.

[169] Sup SJ, Alemañy CA, Pohlman B (2005). Expression of bcl-2 in classical Hodgkin’s lymphoma: an independent predictor of poor outcome. *Journal of Clinical Oncology*.

[170] Brink AATP, Oudejans JJ, van den Brule AJC (1998). Low p53 and high bcl-2 expression in Reed-Sternberg cells predicts poor clinical outcome for Hodgkin’s disease: involvement of apoptosis resistance?. *Modern Pathology*.

[171] Garcia JF, Camacho FI, Morente M (2003). Hodgkin and Reed-Sternberg cells harbor alterations in the major tumor suppressor pathways and cell-cycle checkpoints: analyses using tissue microarrays. *Blood*.

[172] Smolewski P, Robak T, Krykowski E (2000). Prognostic factors in Hodgkin’s disease: multivariate analysis of 327 patients from a single institution. *Clinical Cancer Research*.

[173] Abele MC, Valente G, Kerim S (1997). Significance of cell proliferation index in assessing histological prognostic categories in Hodgkin’s disease: an immunohistochemical study with Ki67 and MIB-1 monoclonal antibodies. *Haematologica*.

[174] Bai M, Papoudou-Bai A, Kitsoulis P (2005). Cell cycle and apoptosis deregulation in classical Hodgkin lymphomas. *In Vivo*.

[175] Devilard E, Bertucci F, Trempat P (2002). Gene expression profiling defines molecular subtypes of classical Hodgkin’s disease. *Oncogene*.

[176] Famularo G, De Simone C, Tzantzoglou S, Trinchieri V (1994). Apoptosis, anti-apoptotic compounds and TNF-*α* release. *Immunology Today*.

[177] Hahne M, Renno T, Schroeter M (1996). Activated B cells express functional Fas ligand. *European Journal of Immunology*.

[178] Álvaro T, Lejeune M, García JF (2008). Tumor-infiltrated immune response correlates with alterations in the apoptotic and cell cycle pathways in Hodgkin and Reed-Sternberg cells. *Clinical Cancer Research*.

[179] Metkar SS, Manna PP, Anand M, Naresh KN, Advani SH, Nadkarni JJ (2001). CD40 ligand—an anti-apoptotic molecule in Hodgkin’s disease. *Cancer Biotherapy and Radiopharmaceuticals*.

[180] Kater AP, Evers LM, Remmerswaal EBM (2004). CD40 stimulation of B-cell chronic lymphocytic leukaemia cells enhances the anti-apoptotic profile, but also Bid expression and cells remain susceptible to autologous cytotoxic T-lymphocyte attack. *British Journal of Haematology*.

[181] Inoue JI, Ishida T, Tsukamoto N (2000). Tumor necrosis factor receptor-associated factor (TRAF) family: adapter proteins that mediate cytokine signaling. *Experimental Cell Research*.

[182] Lee HH, Dadgostar H, Cheng Q, Shu J, Cheng G (1999). NF-*κ*B-mediated up-regulation of Bcl-x and Bfl-1/A1 is required for CD40 survival signaling in B lymphocytes. *Proceedings of the National Academy of Sciences of the United States of America*.

[183] Hong SY, Yoon WH, Park JH, Kang SG, Ahn JH, Lee TH (2000). Involvement of two NF-*κ*B binding elements in tumor necrosis factor *α*-, CD40-, and Epstein-Barr virus latent membrane protein 1-mediated induction of the cellular inhibitor of apoptosis protein 2 gene. *Journal of Biological Chemistry*.

[184] Thome M, Tschopp J (2001). Regulation of lymphocyte proliferation and death by flip. *Nature Reviews Immunology*.

[185] Malumbres M, Barbacid M (2001). To cycle or not to cycle: a critical decision in cancer. *Nature Reviews Cancer*.

[186] Wagner EF, Hieb M, Hanna N, Sharma S (1998). A pivotal role of cyclin D3 and cyclin-dependent kinase inhibitor p27 in the regulation of IL-2-, IL-4-, or IL-10-mediated human B cell proliferation. *Journal of Immunology*.

[187] Blanchard DA, Affredou MT, Vazquez A (1997). Modulation of the p27kip1 cyclin-dependent kinase inhibitor expression during IL-4-mediated human B cell activation 1. *Journal of Immunology*.

[188] Ren F, Zhan X, Martens G (2005). Pro-IL-16 regulation in activated murine CD4^+^ lymphocytes. *Journal of Immunology*.

[189] Shaffer AL, Yu X, He Y, Boldrick J, Chan EP, Staudt LM (2000). BCL-6 represses genes that function in lymphocyte differentiation, inflammation, and cell cycle control. *Immunity*.

[190] Tang TTL, Dowbenko D, Jackson A (2002). The forkhead transcription factor AFX activates apoptosis by induction of the BCL-6 transcriptional repressor. *Journal of Biological Chemistry*.

[191] Calò V, Migliavacca M, Bazan V (2003). STAT proteins: from normal control of cellular events to tumorigenesis. *Journal of Cellular Physiology*.

[192] Rudant J, Orsi L, Monnereau A (2011). Childhood hodgkin's lymphoma, non-Hodgkin's lymphoma and factors related to the immune system: the Escale study (SFCE). *International Journal of Cancer*.

[193] Khan G (2006). Epstein-Barr virus, cytokines, and inflammation: a cocktail for the pathogenesis of Hodgkin’s lymphoma?. *Experimental Hematology*.

[194] Kapatai G, Murray P (2007). Contribution of the Epstein-Barr virus to the molecular pathogenesis of Hodgkin lymphoma. *Journal of Clinical Pathology*.

[195] Jarrett RF (2002). Viruses and Hodgkin’s lymphoma. *Annals of Oncology*.

[196] Jarrett RF (2006). Viruses and lymphoma/leukaemia. *Journal of Pathology*.

[197] Lam N, Sugden B (2003). CD40 and its viral mimic, LMP1: similar means to different ends. *Cellular Signalling*.

[198] Kilger E, Kieser A, Baumann M, Hammerschmidt W (1998). Epstein-Barr virus-mediated B-cell proliferation is dependent upon latent membrane protein 1, which simulates an activated CD40 receptor. *The EMBO Journal*.

[199] Young LS, Rickinson AB (2004). Epstein-Barr virus: 40 years on. *Nature Reviews Cancer*.

[200] Kennedy G, Komano J, Sugden B (2003). Epstein-Barr virus provides a survival factor to Burkitt’s lymphomas. *Proceedings of the National Academy of Sciences of the United States of America*.

[201] Aldinucci D, Lorenzon D, Cattaruzza L (2008). Expression of CCR5 receptors on Reed-Sternberg cells and Hodgkin lymphoma cell lines: involvement of CCL5/Rantes in tumor cell growth and microenvironmental interactions. *International Journal of Cancer*.

[202] Fischer M, Juremalm M, Olsson N (2003). Expression of CCL5/RANTES by Hodgkin and reed-sternberg cells and its possible role in the recruitment of mast cells into lymphomatous tissue. *International Journal of Cancer*.

[203] Hanamoto H, Nakayama T, Miyazato H (2004). Expression of CCL28 by reed-sternberg cells defines a major subtype of classical Hodgkin’s disease with frequent infiltration of eosinophils and/or plasma cells. *American Journal of Pathology*.

[204] Baumforth KRN, Birgersdotter A, Reynolds GM (2008). Expression of the Epstein-Barr virus-encoded Epstein-Barr virus nuclear antigen 1 in Hodgkin’s lymphoma cells mediates up-regulation of CCL20 and the migration of regulatory T cells. *American Journal of Pathology*.

[205] Niens M, Visser L, Nolte IM (2008). Serum chemokine levels in Hodgkin lymphoma patients: highly increased levels of CCL17 and CCL22. *British Journal of Haematology*.

[206] Chetaille B, Bertucci F, Finetti P (2009). Molecular profiling of classical Hodgkin lymphoma tissues uncovers variations in the tumor microenvironment and correlations with EBV infection and outcome. *Blood*.

[207] Frisan T, Sjoberg J, Dolcetti R (1995). Local suppression of Epstein-Barr virus (EBV)-specific cytotoxicity in biopsies of EBV-positive Hodgkin’s disease. *Blood*.

[208] Bosch Príncep R, Lejeune M, Salvadó Usach MT, Jaén Martínez J, Pons Ferré LE, Álvaro Naranjo T (2005). Decreased number of granzyme B^+^ activated CD8^+^ cytotoxic T lymphocytes in the inflammatory background of HIV-associated Hodgkin’s lymphoma. *Annals of Hematology*.

[209] Ladoire S, Martin F, Ghiringhelli F (2011). Prognostic role of FOXP3+ regulatory T cells infiltrating human carcinomas: the paradox of colorectal cancer. *Cancer Immunology, Immunotherapy*.

[210] Marshall NA, Culligan DJ, Tighe J, Johnston PW, Barker RN, Vickers MA (2007). The relationships between Epstein-Barr virus latent membrane protein 1 and regulatory T cells in Hodgkin’s lymphoma. *Experimental Hematology*.

[211] Zitvogel L, Apetoh L, Ghiringhelli F, André F, Tesniere A, Kroemer G (2008). The anticancer immune response: indispensable for therapeutic success?. *Journal of Clinical Investigation*.

[212] Casares N, Pequignot MO, Tesniere A (2005). Caspase-dependent immunogenicity of doxorubicin-induced tumor cell death. *Journal of Experimental Medicine*.

[213] Nowak AK, Robinson BWS, Lake RA (2003). Synergy between chemotherapy and immunotherapy in the treatment of established murine solid tumors. *Cancer Research*.

[214] Haynes NM, van der Most RG, Lake RA, Smyth MJ (2008). Immunogenic anti-cancer chemotherapy as an emerging concept. *Current Opinion in Immunology*.

[215] Tesniere A, Apetoh L, Ghiringhelli F (2008). Immunogenic cancer cell death: a key-lock paradigm. *Current Opinion in Immunology*.

[216] Peggs KS, Quezada SA, Chambers CA, Korman AJ, Allison JP (2009). Blockade of CTLA-4 on both effector and regulatory T cell compartments contributes to the antitumor activity of anti-CTLA-4 antibodies. *Journal of Experimental Medicine*.

[217] Attia P, Maker AV, Haworth LR, Rogers-Freezer L, Rosenberg SA (2005). Inability of a fusion protein of IL-2 and diphtheria toxin (Denileukin Diftitox, DAB389IL-2, ONTAK) to eliminate regulatory T lymphocytes in patients with melanoma. *Journal of Immunotherapy*.

[218] de la Cruz-Merino L, Grande-Pulido E, Albero-Tamarit A, De Villena MECM (2008). Cancer and immune response: old and new evidence for future challenges. *Oncologist*.

[219] Suzuki E, Kapoor V, Jassar AS, Kaiser LR, Albelda SM (2005). Gemcitabine selectively eliminates splenic Gr-1^+^/CD11b^+^ myeloid suppressor cells in tumor-bearing animals and enhances antitumor immune activity. *Clinical Cancer Research*.

[220] Lake RA, Robinson BWS (2005). Immunotherapy and chemotherapy—a practical partnership. *Nature Reviews Cancer*.

[221] Lake RA, van der Most RG (2006). A better way for a cancer cell to die. *The New England Journal of Medicine*.

[222] Formenti SC, Demaria S (2009). Systemic effects of local radiotherapy. *The Lancet Oncology*.

[223] Mantovani A, Allavena P, Sica A, Balkwill F (2008). Cancer-related inflammation. *Nature*.

[224] Reits EA, Hodge JW, Herberts CA (2006). Radiation modulates the peptide repertoire, enhances MHC class I expression, and induces successful antitumor immunotherapy. *Journal of Experimental Medicine*.

[225] Coates PJ, Rundle JK, Lorimore SA, Wright EG (2008). Indirect macrophage responses to ionizing radiation: implications for genotype-dependent bystander signaling. *Cancer Research*.

[226] Wright EG, Coates PJ (2006). Untargeted effects of ionizing radiation: implications for radiation pathology. *Mutation Research*.

[227] Apetoh L, Ghiringhelli F, Tesniere A (2007). Toll-like receptor 4-dependent contribution of the immune system to anticancer chemotherapy and radiotherapy. *Nature Medicine*.

[228] Antoniades J, Brady LW, Lightfoot DA (1977). Lymphangiographic demonstration of the abscopal effect in patients with malignant lymphomas. *International Journal of Radiation Oncology Biology Physics*.

[229] Ohba K, Omagari K, Nakamura T (1998). Abscopal regression of hepatocellular carcinoma after radiotherapy for bone metastasis. *Gut*.

[230] Rees GJG, Ross CMD (1983). Abscopal regression following radiotherapy for adenocarcinoma. *British Journal of Radiology*.

[231] Camphausen K, Moses MA, Ménard C (2003). Radiation abscopal antitumor effect is mediated through p53. *Cancer Research*.

[232] Abbas A, Lichtman A, Pillai S (2007). Immunity to tumors. *Cellular and Molecular Immunology*.

[233] Sharpe AH, Abbas AK (2006). T-cell costimulation—biology, therapeutic potential, and challenges. *The New England Journal of Medicine*.

[234] Carreno BM, Bennett F, Chau TA (2000). CTLA-4 (CD152) can inhibit T cell activation by two different mechanisms depending on its level of cell surface expression. *Journal of Immunology*.

[235] Robert C, Ghiringhelli F (2009). What is the role of cytotoxic T lymphocyte-associated antigen 4 blockade in patients with metastatic melanoma?. *Oncologist*.

[236] Hodi FS, O’Day SJ, McDermott DF (2010). Improved survival with ipilimumab in patients with metastatic melanoma. *The New England Journal of Medicine*.

[237] Robert C, Thomas L, Bondarenko I (2011). Ipilimumab plus dacarbazine for previously untreated metastatic melanoma. *The New England Journal of Medicine*.

[238] Bashey A, Medina B, Corringham S (2009). CTLA4 blockade with ipilimumab to treat relapse of malignancy after allogeneic hematopoietic cell transplantation. *Blood*.

[239] Azuma T, Yao S, Zhu G, Flies AS, Flies SJ, Chen L (2008). B7-H1 is a ubiquitous antiapoptotic receptor on cancer cells. *Blood*.

[240] Parekh VV, Lalani S, Kim S (2009). PD-1/PD-L blockade prevents anergy induction and enhances the anti-tumor activities of glycolipid-activated invariant NKT cells. *Journal of Immunology*.

[241] Aldinucci D, Gloghini A, Pinto A, Colombatti A, Carbone A (2012). The role of CD40/CD40L and interferon regulatory factor 4 in Hodgkin lymphoma microenvironment. *The Leukemia & Lymphoma*.

[242] Fonsatti E, Maio M, Altomonte M, Hersey P (2010). Biology and clinical applications of CD40 in cancer treatment. *Seminars in Oncology*.

[243] Aldinucci D, Rapana’ B, Olivo K (2010). IRF4 is modulated by CD40L and by apoptotic and anti-proliferative signals in Hodgkin lymphoma. *British Journal of Haematology*.

[244] Advani R, Forero-Torres A, Furman RR (2009). Phase I study of the humanized anti-CD40 monoclonal antibody dacetuzumab in refractory or recurrent non-Hodgkin’s lymphoma. *Journal of Clinical Oncology*.

[245] Ansell SM, Hurvitz SA, Koenig PA (2009). Phase I study of ipilimumab, an anti-CTLA-4 monoclonal antibody, in patients with relapsed and refractory B-cell non-Hodgkin lymphoma. *Clinical Cancer Research*.

[246] Younes A, Oki Y, McLaughlin P (2012). Phase 2 study of rituximab plus ABVD in patients with newly diagnosed classical Hodgkin lymphoma. *Blood*.

[247] Younes A, Romaguera J, Hagemeister F (2003). A pilot study of rituximab in patients with recurrent, classic Hodgkin disease. *Cancer*.

[248] Oki Y, Pro B, Fayad LE (2008). Phase 2 study of gemcitabine in combination with rituximab in patients with recurrent or refractory Hodgkin lymphoma. *Cancer*.

[249] Younes A, Wong F (2009). Experience with 90Y-ibritumomab tiuxetan for relapsed classical Hodgkin lymphoma. *Annals of Oncology*.

[250] Ambrose LR, Morel AS, Warrens AN (2009). Neutrophils express CD52 and exhibit complement-mediated lysis in the presence of alemtuzumab. *Blood*.

[251] Peggs KS, Sureda A, Qian W (2007). Reduced-intensity conditioning for allogeneic haematopoietic stem cell transplantation in relapsed and refractory Hodgkin lymphoma: impact of alemtuzumab and donor lymphocyte infusions on long-term outcomes. *British Journal of Haematology*.

[252] Fehniger TA, Larson S, Trinkaus K (2011). A phase 2 multicenter study of lenalidomide in relapsed or refractory classical Hodgkin lymphoma. *Blood*.

[253] Chanan-Khan AA, Cheson BD (2008). Lenalidomide for the treatment of B-cell malignancies. *Journal of Clinical Oncology*.

[254] Corazzelli G, De Filippi R, Capobianco G (2010). Tumor flare reactions and response to lenalidomide in patients with refractory classic Hodgkin lymphoma. *American Journal of Hematology*.

[255] Fehniger T, Larson S, Trinkaus K (2009). A phase II multicenter study of lenalidomide in relapsed or refractory classical Hodgkin lymphoma. *Blood*.

[256] Kuruvilla J, Taylor D, Wang L, Blattler C, Keating A, Crump M (2008). Phase II trial of lenalidomide in patients with relapsed or refractory Hodgkin lymphoma. *Blood*.

[257] Younes A, Bartlett NL, Leonard JP (2010). Brentuximab vedotin (SGN-35) for relapsed CD30-positive lymphomas. *The New England Journal of Medicine*.

[258] Gualberto A (2012). Brentuximab Vedotin (SGN-35), an antibody-drug conjugate for the treatment of CD30-positive malignancies. *Expert Opinion on Investigational Drugs*.

[259] Redmond WL, Ruby CE, Weinberg AD (2009). The Role of OX40-mediated Co-stimulation in T-cell activation and survival. *Critical Reviews in Immunology*.

[260] Jensen SM, Maston LD, Gough MJ (2010). Signaling through OX40 enhances antitumor immunity. *Seminars in Oncology*.

[261] Buglio D, Khaskhely NM, Voo KS, Martinez-Valdez H, Liu YJ, Younes A (2011). HDAC11 plays an essential role in regulating OX40 ligand expression in Hodgkin lymphoma. *Blood*.

[262] Buglio D, Younes A (2010). Histone deacetylase inhibitors in Hodgkin lymphoma. *Investigational New Drugs*.

[263] Younes A, Wong F (2009). Experience with 90Y-ibritumomab tiuxetan for relapsed classical Hodgkin lymphoma. *Annals of Oncology*.

[264] Hallek M, Cheson BD, Catovsky D (2008). Guidelines for the diagnosis and treatment of chronic lymphocytic leukemia: a report from the International Workshop on Chronic Lymphocytic Leukemia updating the National Cancer Institute-Working Group 1996 guidelines. *Blood*.

[265] Böll B, Borchmann P, Topp MS (2010). Lenalidomide in patients with refractory or multiple relapsed Hodgkin lymphoma. *British Journal of Haematology*.

[266] Deutsch YE, Tadmor T, Podack ER, Rosenblatt JD (2011). CD30: an important new target in hematologic malignancies. *The Leukemia & Lymphoma*.

[267] Okeley NM, Miyamoto JB, Zhang X (2010). Intracellular activation of SGN-35, a potent anti-CD30 antibody-drug conjugate. *Clinical Cancer Research*.

